# A Cancer-Specific Qualitative Method for Estimating the Proportion of Tumor-Infiltrating Immune Cells

**DOI:** 10.3389/fimmu.2021.672031

**Published:** 2021-05-14

**Authors:** Huiting Xiao, Jiashuai Zhang, Kai Wang, Kai Song, Hailong Zheng, Jing Yang, Keru Li, Rongqiang Yuan, Wenyuan Zhao, Yang Hui

**Affiliations:** ^1^ Department of Systems Biology, College of Bioinformatics Science and Technology, Harbin Medical University, Harbin, China; ^2^ Department of Biochemistry and Molecular Biology, Harbin Medical University, Harbin, China

**Keywords:** tumor microenvironment, relative expression orderings, signature genes, prognosis, immunotherapy

## Abstract

Tumor-infiltrating immune cells are important components in the tumor microenvironment (TME) and different types of these cells exert different effects on tumor development and progression; these effects depend upon the type of cancer involved. Several methods have been developed for estimating the proportion of immune cells using bulk transcriptome data. However, there is a distinct lack of methods that are capable of predicting the immune contexture in specific types of cancer. Furthermore, the existing methods are based on absolute gene expression and are susceptible to experimental batch effects, thus resulting in incomparability across different datasets. In this study, we considered two common neoplasms as examples (colorectal cancer [CRC] and melanoma) and introduced the Tumor-infiltrating Immune Cell Proportion Estimator (TICPE), a cancer-specific qualitative method for estimating the proportion of tumor-infiltrating immune cells. The TICPE was based on the relative expression orderings (REOs) of gene pairs within a sample and is notably insensitive to batch effects. Performance evaluation using public expression data with mRNA mixtures, single-cell RNA-Seq (scRNA-Seq) data, immunohistochemistry data, and simulated bulk RNA-seq samples, indicated that the TICPE can estimate the proportion of immune cells with levels of accuracy that are clearly superior to other methods. Furthermore, we showed that the TICPE could effectively detect prognostic signals in patients with tumors and changes in the fractions of immune cells during immunotherapy in melanoma. In conclusion, our work presented a unique novel method, TICPE, to estimate the proportion of immune cells in specific cancer types and explore the effect of the infiltration of immune cells on the efficacy of immunotherapy and the prognosis of cancer. The source code for TICPE is available at https://github.com/huitingxiao/TICPE.

## Introduction

Immune cells are critical components in the complex tumor environment (TME). Tumor-infiltrating immune cells (TIICs) can have either tumor-promoting or tumor-suppressive effects on tumor development and progression, depending on the specific type of cancer involved (1). The types and densities of TIICs not only have predictive value in patient survival, they also affect tumor responses to therapy, particularly immunotherapy (2, 3). For instance, an increase in CD8+ T cells is generally associated with improved clinical outcomes, whereas regulatory T cells (Tregs) and tumor-associated macrophages (TAMs) are often associated with a poor prognosis (4, 5). In addition, immune checkpoint blockade (ICB) antibodies reinvigorate anti-tumor immunotherapy responses by disrupting co-inhibitory T-cell signaling, a pathway that has demonstrated clinical activity in several malignancies (6). Evidence has shown that CD4+ and CD8+ memory T cell subsets, as well as NK cell subsets, correlated with a clinical response to immunotherapy in patients with melanoma (7, 8). As such, an assessment of TIICs is of critical importance in biomedical research as well as clinical pathology (9).

Previous studies concerning alterations in the composition of immune cells in human cancers have predominantly relied on immunohistochemistry (IHC) or flow cytometry. However, these techniques are compromised by the limited set of available molecular markers and are cumbersome to apply to large panels of tumors; furthermore, in the case flow cytometry, fresh or frozen tissue is required (10, 11). An abundance of transcriptomics data provide an ideal resource for large-scale immune landscape analysis and have been used to develop many computational methods that have been mainly classified into two categories: deconvolution-based approaches and methods that are based on marker genes (12). The deconvolution methods, which include CIBERSORT (13), TIMER (14), EPIC (15), and quanTIseq (16), estimate the cell fractions leveraging on a reference matrix composed of representative expression signatures for specific immune cells. Techniques that are based on marker genes, including MCP-counter (17), xCell (18), and ImmuCellAI (19), utilize a list of genes characterized for each immune cell type to compute an enrichment score and allow for inter-sample comparisons of the same immune cell type. However, these methods have been developed for the enumeration of immune cells from bulk transcriptome data from multiple cancer types that masked inter-tumor heterogeneity between different tumor types; this would affect accuracy, at least to some extent (20). In addition, all of these methods were based on absolute gene expression, thus resulting in incomparability across different datasets. Some of these techniques require data normalization, a process that is susceptible to experimental batch effects and can even distort real biological signals (21). In contrast, our research team has proven that qualitative information derived from relative gene expression is highly robust with regards to batch effects and does not necessarily require normalization (22, 23). It is therefore imperative to develop a cancer-specific qualitative method to estimate the proportion of tumor-infiltrating immune cells.

In this study, we considered colorectal cancer and melanoma as examples and constructed Tumor-infiltrating Immune Cell Proportion Estimator (TICPE), a qualitative method based on the relative expression orderings (REOs) of gene pairs within a sample, to estimate the proportion of immune cells in a TME. These cell proportions could then be used to directly compare the proportion of the corresponding immune cells across samples within a cohort or different cohorts. TICPE was extensively validated in human solid tumors *via* publicly available IHC data, mRNA mixtures, single-cell RNA-Seq (scRNA-Seq) data from colorectal and melanoma tumors, and simulated bulk samples. Moreover, the immune cell proportions estimated by TICPE could be used for prognostic analysis and associated with treatment status and the efficacy of immunotherapy response to melanoma.

## Materials and Methods

### Dataset Preparation

We downloaded gene expression datasets from the Gene Expression Omnibus (GEO, http://cancergenome.nih.gov/) and RNA sequencing data from The Cancer Genome Atlas (TCGA) by the University of California Santa Cruz (UCSC) Xena website (https://xena.ucsc.edu/). The processed gene expression profiles of 97 datasets were divided into three sections (see **Supplementary Table 1**). Eighty-one of these datasets contained eight types of human immune cells and cancer cell lines; normal samples were used to generate signature genes and develop the TICPE. Five datasets were used for to assess the performance of the TICPE. Dataset 1 was derived from an *in vitro* RNA mixture experiment, GEO accession GSE64385. These mixtures contained different immune populations that were purified from the peripheral blood from healthy donors with variable concentrations and were further diluted in a fixed amount of a solution containing mRNA extracted from HCT-116, a CRC cell line (**Supplementary Table 2A**). Dataset 2 contained a large series of 566 CRC tumors and 19 non-tumoral colorectal mucosas, GEO accession GSE39582. Of the 566 tumors, 33 patients also had immunohistochemistry data relating to CD3, CD8, and CD68 (Aurélien de Reyniès, Personal Communication). The other three datasets (Accession numbers GSE146771, GSE115978, and GSE72056) were scRNA-Seq data, and corresponded to 10 colon cancer samples, and 31 and 19 patients with melanoma, respectively (see **Supplementary Tables 2B–D**). The remaining datasets that were associated with clinical information were used to investigate prognosis and response to immunotherapy. The response categories of the melanoma patients were defined by the RECIST classification scheme (Response Evaluation Criteria in Solid Tumors) as a complete response (CR) and partial response (PR) for responders, or stable disease (SD) and progressive disease (PD) for non-responders (24).

For the data downloaded from GEO, we mapped the probe ID to the Entrez gene ID using the corresponding platform annotation file. Data were discarded if a probe had no or multiple corresponding Entrez gene IDs. If multiple probes shared the same Entrez gene ID, then the arithmetic mean of the expression values of these probes was used as the final expression value of the gene. For the RNA-Seq data, profiles of fragments per kilobase million (FPKM) were directly downloaded from the TCGA. For scRNA-Seq data, the reconstructed bulk samples from each donor were identified by aggregating expression profiles from all cell barcodes of the given donor. The cell ratio per cell type in a donor was then calculated by the cell number of a specific cell type divided by the total number of cells (19).

### Marker Gene Preparation

For each immune cell type, we integrated a list of marker genes obtained from the literature and other analytical methods, such as xCell and MCP-counter. Most of these were overexpressed relative to other immune cells, and a total of 2,034 marker genes were acquired (see **Supplementary Table 3**).

### Highly Stable Pairs in Cancer Cell Lines

For each cancer cell, pairwise comparisons were performed for the expression level of all genes. For each gene pair (*G_i_*, *G_j_*), with only two possible REO outcomes (the gene expression of *G_i_* > *G_j_* or *G_i_* < *G_j_*), we retained the gene pair with a certain REO (*G_i_* > *G_j_* or *G_i_* < *G_j_*) in at least 99% cancer cells, defined as a highly stable gene pair (SPairs).

### The TICPE Development Pipeline

**Figure 1:**
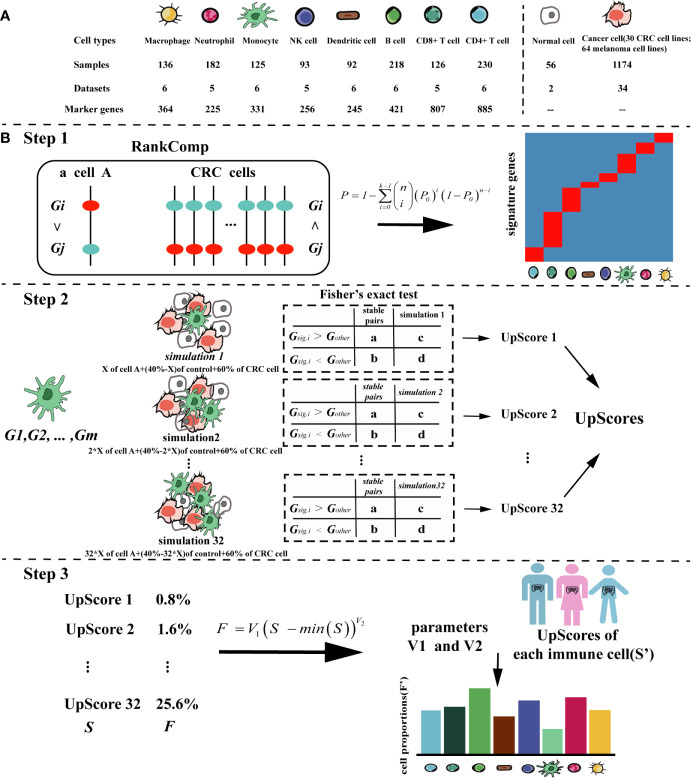
The pipeline of the TICPE algorithm. **(A)** Summary of the data sources used in the study to develop the TICPE. **(B)** The pipeline of the TICPE algorithm. RankComp algorithm was used to identify robust signature genes compared with cancer cells for each type of immune cell from the known marker genes. The upregulated score was the reversal significance of all signature genes corresponding to the cell type. Using the simulated model for each cell type, we derived a transformation pipeline for the scores. For each queried sample, calculated upregulated scores and transformed them to estimated cell proportions using learned parameters.

This cancer-specific method can be used for a variety of cancer types. Here, we took colorectal cancer as an example to describe the process in detail, the flowchart for TICPE is described in **Figure 1**.

#### Identifying Signature Genes Compared With CRC Cells

Since cell-type-specific signatures can vary depending on cancer type, there is a need to identify cancer-type specific marker genes for each immune cell type. We take immune cell type *A* with *n* cells as an example to illustrate the process that can be used to identify the corresponding signature genes among reported marker genes. Firstly, we used the RankComp algorithm (25) to identify individual-level differentially upregulated marker genes (up-DEGs) for each immune cell compared with the SPairs in the CRC cell lines. The p-values of RankComp were adjusted using the Benjamini and Hochberg method (26). Secondly, the cumulative binomial distribution model was used to identify up-DEGs shared by a non-random high proportion of samples and the *P* value determined whether a marker gene was differentially upregulated at the population level. Then, the *P* values were also adjusted for multiple testing to control the false discovery rate (FDR). The significance was calculated as shown in Equation (1).

Equation (1):

$$P=1-\sum_{i=0}^{k-1}\left(\begin{array}{l} n\\ i \end{array}\right){\left(P_{0}^{}\right)}^{i}{\left(1-P_{0}\right)}^{n-i}$$

In Equation (1), *P_0_* represents the probability of observing a marker gene being differentially upregulated in a sample by chance (*P_0_* = 0.5), *n* and *k* represent the total number of samples of the immune cell type *A* and the number of samples with the marker gene being differentially upregulated, respectively. Next, when a marker gene’s adjusted P-value was <0.05, it would be reserved. We finally removed the up-DEGs included in more than one type of immune cell to reduce the dependencies between closely related cell types, and the remained up-DEGs were defined as signature genes (Step 1).

#### Calculating Upregulated Scores Based on Signature Genes

Based on the signature genes for each immune cell type, we were able to compute the cell infiltration scores for each sample. However, the scores had different distributions between different signature genes and could not thus be compared across immune cell types in a sample. Thus, for each cell type, we conducted a simulated model using the immune cell (cell *A*) with an additional “control” cell type (a sample of normal colon) and a variety of CRC cell lines. Different types of CRC cell lines were used to reflect the heterogeneity of patients with the same cancer. For the simulated models, batch effects among the three types of dataset were removed using Combat (27). Then, we generated such simulations by using the median expression profile of the merged profile composed of three cell types: 60% of the CRC cell, X% of cell *A*, and 40–X% control (28). X% represents an arithmetic sequence with a range of 0.8 to 25.6% and an interval of 0.8%. We used this range because these interesting cell types had low fractions in the TME (18).

Taking the SPairs of CRC cell lines as the background, we calculated the cell infiltration scores of the simulations using *m* signature genes {*G_sig.1_*, *G_sig.2_*,… *G_sig.m_*}, as described below. We were able to calculate the numbers of gene pairs (the gene pair was constructed by *G_sig_* and *G_other_*) belonging to SPairs with ordering patterns (*G_sig.i_* > *G_other_*) and (*G_sig.i_* < *G_other_*) in cancer cells, which were denoted *a* and *b*. Similarly, *c* and *d* denoted the corresponding numbers of gene pairs with ordering patterns (*G_sig.i_* > *G_other_*) and (*G_sig.i_* < *G_other_*) in a simulation sample. When simulations involved an increasing proportion of immune cells, there were more stable pairs with the ordering patterns (*G_sig.i_* > *G_other_*). Using Fisher’s exact test, we were able to determine the degree of cell infiltration by calculating the reversal significance, also known as the upregulated score (UpScore) for each simulation sample (Step 2).

#### Transforming UpScores to Estimate Cell Proportions

We designed a transformation pipeline for the UpScores of each cell type to enable the estimated proportions to be compared across cell types, and not just across samples. A simulation containing 0.8% of immune cells was considered to barely result in reversal significance. Therefore, for the simulated model of cell *A*, we first shifted the UpScores to 0 using the minimal UpScore (which corresponded to the simulation containing 0.8% of cell *A*) and fitted a power function to the UpScores that corresponded to proportions of 0.8 to 25.6%. The transformed parameters (*V_1_* and *V_2_*) were acquired by Equation (2). For each immune cell type, we could get a pair of transformed parameters.

Equation (2):

$$F_{i}=V_{1i}{\left(S_{i}-\min\left(S_{i}\right)\right)}^{V_{2i}}$$

In Equation (2), *F* represents the proportions of 0.8 to 25.6% and *S* represents the corresponding UpScores of cell *A*.

It was recommended that an expression dataset should contain as many signature genes as possible. The UpScores of different immune cell types were calculated based on their different signature genes. Subsequently, using the parameters corresponding to immune cells, the UpScores were transformed into estimated cell proportions for each sample (Step 3).

### The Generation of Simulated Data

We simulated bulk RNA-seq data with different tumor purity values and immune infiltrates by mixing malignant cells with different immune cells from a scRNA-seq dataset. There were 100 simulated samples and each of these was composed of 1,000 cells that were randomly selected, as follows: (i) Cancer cells form the majority of a simulated samples, a fraction *f* of them was constrained to the interval [0.5, 0.99], and the remaining fraction *1 − f* was randomly assigned to the other immune cell types; (ii) the fraction was multiplied by 1,000 to obtain cell counts for different cell types; (iii) the corresponding number of cells was randomly selected from the single cell dataset. If one cell type was available from the scRNA-seq dataset with only a few cells available, then, the same single cell sample would be selected multiple times for the artificial bulk sample.

### Performance Assessment of the TICPE

The performance of the TICPE was evaluated using both microarray and RNA-Seq datasets and compared with that of previously published methods (CIBERSORT, EPIC, xCell, MCP-counter, and ImmuCellAI). For a given immune cell type, the accuracy and sensitivity of each method were measured using Pearson’s correlation between the results of *in silico* methods and the true proportions, as measured by immunohistochemistry or scRNA-Seq. Furthermore, we introduced a correlation deviation (19) for all cell types to measure the global performance of each method; this strategy took the sample size and overall accuracy into consideration. A smaller correlation deviation might suggest that the predicted cell fractions agree better with the true composition, as shown in Equation (3).

Equation (3):

$$\text{correlation deviation}={\sqrt{\frac{1}{n}\sum_{i=1}^{n}{\left(1-r_{i}\right)}^{2}}}^{}$$

In Equation (3), *n* represents the number of immune cell types detected in samples and *r_i_* represents the Pearson correlation coefficient of immune cell type *i*.

### Statistical Analysis

The correlation between estimated proportion and true composition was evaluated by Pearson’s correlation. The ROC analysis was performed to assess the validity of the TICPE and was completed by pROC R Package. The statistical significance of comparisons between two groups or more than two groups was estimated by the Wilcoxon rank-sum test or the Kruskal–Wallis test, respectively. The overall survival curves were estimated by the Kaplan–Meier method, and the differences between survival distributions were evaluated by the two-sided log-rank test (29). The Venn diagram was used to analyze the signature genes were different between CRC and melanoma by ggvenn R Package. All statistical analyses were performed using R program (version 4.0.2). P-values were two-sided, and *P <*0.05 was considered to be statistically significant (4).

## Results

### Development of the TICPE Algorithm

We designed a method, called TICPE, to estimate the proportions of eight important tumor-infiltrating immune cells (B cells, CD4^+^ T cells, CD8^+^ T cells, dendritic cells (DC), monocytes, macrophages, natural killer (NK) cells, and neutrophils) in a specify type of cancer. We integrated marker genes for these different cell types from publications and obtained expression profiles for immune cells, cancer cell lines, and normal tissues, from the GEO database (**Figure 1A**; **Table 1**). Taking colorectal cancer as an example, a three-step strategy of the core algorithm of TICPE is shown in **Figure 1B**; the detailed algorithm is described in the *Materials and Methods* section. Since the marker genes were screened relative to other immune cells, but not to tumor cells, the genes for a specific type of cancer needed to be filtered. We applied the RankComp algorithm to detect overexpressed marker genes in a given immune cell compared with CRC cells (*FDR <*5%). We then created a specific gene set from overexpressed marker genes as signature genes; this included 218 genes from the eight immune cell types (Binomial test, *FDR <*5%) (**Table 2**) (Step 1). We were then able to compute the cell infiltration scores for each sample based on the signature genes (Fisher’s exact test) (Step 2). However, the scores exhibited different distributions between different signature genes and could not therefore be compared across cell types in a given sample. For each immune cell type, we thus conducted a simulated model using the immune cells, CRC cells, and normal colon samples, and calculated the UpScores based on signature genes. Next, we designed a transformation pipeline for the UpScores and acquired a pair of transformed parameters for each cell type. For CRC samples, we were able to calculate the UpScores for each immune cell type and transform these into the estimated cell proportions with the acquired parameters corresponding to immune cells (Step 3).

**Table 1:** Datasets used in developing TICPE for colorectal cancer.

Cell Type	Accession	Samples#	Marker Gene#
CRC cells	GSE11618, GSE13059, GSE110425, GSE14103, GSE16648, GSE122985, GSE18560, GSE24795, GSE115716, GSE35566, GSE55624, GSE59196, GSE63252, GSE112282, GSE50841, GSE116528, GSE90085, GSE59883, GSE59857, GSE116529, GSE75205, GSE106073, GSE72544, GSE50791, GSE119197, GSE120993	687	–
B cells	GSE24736, GSE19599, GSE12366, GSE49910, GSE120367, GSE75007	218	422
CD4+ T cells	GSE11292, GSE36769, GSE32959, GSE50175, GSE103527, GSE71956	230	885
CD8+ T cells	GSE84251, GSE93683, GSE98640, GSE84331, GSE71956	126	807
NK cells	GSE27838, GSE8059, GSE21774, GSE35330, GSE75091	93	256
Macrophages	GSE102117, GSE100129, GSE7568, GSE16385, GSE13670, GSE24897	136	364
Monocytes	GSE38351, GSE39840, GSE35683, GSE6054, GSE60199, GSE98480	125	331
DCs	GSE7509, GSE10316, GSE23618, GSE23371, GSE87494, GSE85305	92	245
Neutrophils	GSE22103, GSE39889, GSE8668, GSE18810, GSE70044	182	225

#, number; CRC, colorectal cancer; NK cells, natural killer cells; DCs, dendritic cells.

Table 2AA specific gene set compared with CRC cells for per cell type was selected and used in TICPE.

**Table:** 

Cell Type	Gene Number	Signature Genes
B cells	24	*BLK, CD19, CD79A, CD79B, IGLL1, TCL1A, TLR7, FCRL2, BANK1, CPNE5, KLHL14, LINC00926, FCRL5, EBF1, ARHGAP25, CLECL1, TNFRSF17, FCRLA, HLA-DOB, NCF1, P2RY10, PNOC, TLR9, FCRL4*
CD4+ T cells	65	*ANK1, CD40LG, CD69, CD72, CHI3L2, CCR4, CCR8, DGKA, FYN, GATA3, GPR18, GPR19, IL2RA, IL6R, IL9R, IL12RB2, TNFRSF9, ITGA4, ITGB2, JAK3, LCK, LTB, MAL, CD200, NPAT, P2RX5, PDCD1, PLCL1, PTPRC, RGS1, SELPLG, STAT4, STAT5A, STAT5B, TXK, WIPF1, SOCS3, AIM2, HS3ST3B1, TLR6, CD226, PASK, PLCL2, ANKRD12, STAP1, ZBTB32, LAT, PNMA3, FOXP3, ASB2, LRRN3, LAX1, RNF125, PARP11, PLXDC1, MAN1C1, HIVEP3, BCL11B, PVRIG, ANKRD55, TRIM46, LIMD2, SIGLEC10, RCSD1, PIK3IP1*
CD8+ T cells	42	*ABCD2, RUNX3, CD8A, CD8B, LYST, TSC22D3, GPR183, FLT3LG, HLA-DPB1, IFI16, INPP4A, POU6F1, PTGER4, RAB27A, ATXN7, ZBTB16, EOMES, IL18R1, SLC16A7, ITM2A, AKAP5, TOX, SPOCK2, ZEB2, PLXNC1, CA5B, IKZF2, PTPN22, PBXIP1, IGFLR1, APOL3, KIAA1109, SLA2, SLFN11, JAML, TC2N, TTC39C, TMIGD2, TMEM71, HAPLN3, PYHIN1, JAKMIP1*
DCs	15	*SLAMF8, CCL17, CD1B, CD1E, CLIC2, CD1C, CD209, DNASE1L3, IL3RA, SAMSN1, FABP4, C1QC, SLAMF9, THBD, FAM49A*
Macrophages	18	*CHIT1, CD14, APOC1, HAMP, VSIG4, SDS, SIGLEC7, ADAMDEC1, CCL18, CCL8, CCR1, CMKLR1, CSF1, HS3ST2, MMP19, CPM, ENG, MS4A7*
Monocytes	26	*TLR8, ASGR2, IRAK3, CD33, CFP, CLEC4A, CLEC4E, CXorf21, DOK2, DOK3, FCN1, HCK, LILRA1, LILRA5, LILRB2, MNDA, MYO1F, NCF4, PILRA, PLEK, POU2F2, PSTPIP1, QKI, RETN, CD300LF, SRGN*
NK cells	16	*KIR2DL3, NCR1, FGFBP2, KIR3DS1, PTGDR, LIM2, KIR3DL1, KIR2DS1, KIR3DL3, KIR2DS2, KIR2DS5, KIR2DL1, SH2D1B, KIR2DL4, PIK3CG, KIR2DS4*
Neutrophils	13	*FCGR3B, ALPL, VNN3, FFAR2, MMP25, TREM1, FPR1, LINC00528, CMTM2, PROK2, CLEC7A, CAMP, VNN2*

CRC, colorectal cancer; NK cells, natural killer cells; DCs, dendritic cells.

**Table 2B:** A specific gene set compared with melanoma cells for per cell type was selected.

Cell type	Gene Number	Signature Genes
B cells	15	*MS4A1, BLK, CD19, GNG3, SGCA, CD79A, CD79B*, *CD53, CD72, HTR3A, IGLL1, TCL1A, TLR7, VPREB3*, *AICDA*
CD4+ T cells	85	*LIMD2, TRAF1, NPAT, PIK3IP1, ANKRD12, AAK1*, *ACBD4, CD226, CUBN, GPSM3, GRAP2, IL16, INSL3, JAK3, KLHL3, KRT2, LAIR2, MLH3, MLXIP, NOL9, SELPLG, SORCS3, STAP1, TNK1, TSPAN32, ZNF780B, HS3ST3B1, FOXP3, LAX1, STAT5B, TTN, CCR3, NFATC3, IL2, GGT1, SYNGR3, IL12RB1, STAT4, ZBTB32, CSF2, DPP4, IL12RB2, IL22, EGFL6, IL4, GATA3, IL5, IL13, IL26, ANK1, MB, MICAL2, PHEX, PTGIS, IL1R1, RORC, IL21, IL1R2, IL17A, MAP4K1, SIK1, FOSB, PVRIG, CD69, BCL11B, CHI3L2, DGKA, LAT, LCK, MAP9, PASK, RGS1, SLC7A10, TCF7, TSHR, ZBTB10, TFAP4, COL5A3, ADCYAP1R1, DAB1, ERN1, FXYD7, PNMA3, ARHGEF5, DEFB126*
CD8+ T cells	43	*BLNK, HTR1B, SMCP, RRH, CCDC87, MOGAT2, GJB4, CALY, KIAA1109, CD248, RFX2, AMBN, MYL1, GPR52, CILP, TNFRSF10C, ITGAM, PTGDR2, PRDM1, MPO, RUNX3, APOL3, DUSP2, ZBTB16, CCND2, EOMES, ITM2A, SNX9, CXCL13, HAVCR2, LINC00299, MYO7A, TIGIT, TNFRSF1B, AKAP5, TOX, RGS2, GALM, SYNGR2, PTGER4, CCR6, ATR, GIPR*
DCs	28	*PTGIR, SLAMF8, SLC15A3, SYT17, CCL13, CCL17, CD1B, CD1E, CLIC2, MMP12, TREM2, PLA2G7, ALDH1A2, ALOX15, ALOX15B, BCL2L11, CCL23, CD1A, CD1C, CD209, CD80, DNASE1L3, FLT3, FUT7, GUCA1A, IL12B, IL3RA, KCNK13*
Macrophages	19	*CAMP, CHIT1, CD14, FCGR1A, HAMP, MSR1, VSIG4, SDS, SIGLEC7, TYROBP, ADAMDEC1, CCL7, CCR1, CD84, CMKLR1, CPNE6, CXCL9, CYBA, CYP19A1*
Monocytes	31	*CA1, TLR8, FOLR2, ASGR2, IRAK3, CD33, VCAN, AIF1, CD101, CD93, CEACAM4, CFP, CLEC4A, CXorf21, DOK2, DOK3, FCER1A, FCN1, FGL2, FOLR3, GPR183, HCK, KCNMB1, KDM6B, KSR1, LILRA1, LILRA5, LILRB1, LST1, LY86, LYL1*
NK cells	11	*KIR2DL3, NCR1, NCR3, PRR5L, KIR3DS1, PTGDR, HIPK1, LIM2, NMUR1, PRDM2, TNFSF11*
Neutrophils	19	*CXCR1, FCGR3B, S100A12, TREML2, TRPM6, SIGLEC5, CREB5, ALPL, CEACAM3, VNN3, CA4, CEACAM8, CYP4F3, FFAR2, HBB, MMP25, P2RY13, PGLYRP1, TGM3*

NK cells, natural killer cells; DCs, dendritic cells.

### The Performance of the TICPE in CRC and Melanoma Samples

**Figure 2:**
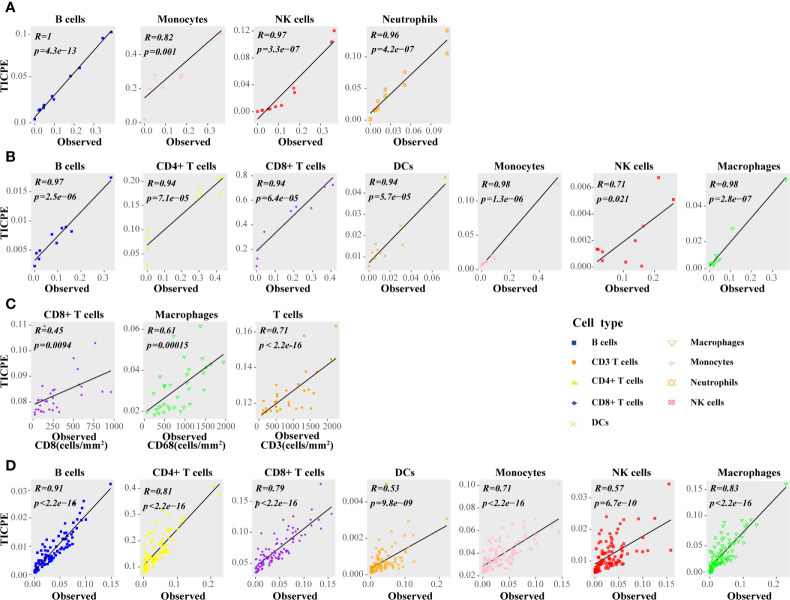
Performance assessment of the TICPE in solid tumors. **(A)** Correlation of the TICPE predictions with the cell proportions for the populations introduced in the mixtures. **(B)** Comparison with single-cell RNA-Seq data from colon samples. **(C)** Correlation of the TICPE predictions with corresponding cell densities measured by immunohistochemistry from colon cancer primary tumors. **(D)** Correlation of the TICPE predictions versus known cell type fractions on 100 simulated bulk samples generated from scRNA-seq from colon samples. Correlations were based on Pearson correlation. Proportions of cells observed experimentally were given in **Supplementary Table 2**.

Firstly, we calculated the proportion of immune cells in CRC samples with three independent publicly available datasets, and simulated RNA-seq data, to evaluate the TICPE. In the *in vitro* RNA mixture experiment (GSE64385), we observed that the estimated cell proportions were highly correlated with the cell proportions for the populations introduced in the mixtures (*ρ *= 0.99 and *P* = 4.2 × 10^−13^ for B cells, *ρ *= 0.82 and *P *= 9.9 × 10^−4^ for monocytes, *ρ *= 0.96 and *P *= 3.3 × 10^−7^ for NK cells, *ρ* = 0.96 and *P *= 4.1 × 10^−7^ for neutrophils; **Figure 2A**). Then, we used scRNA-Seq data from 10 colon cancer samples (GSE146771) and compared TICPE predictions with measured immune cell proportions. As shown in **Figure 2B**, the proportions of immune cells estimated by the TICPE showed a significantly positive correlation with the actual cell proportions (*P <*0.05). We also observed a significant correlation between our predictions and the immunohistochemistry data from 33 colorectal cancer tumors (*P <*0.05; **Figure 2C**). The number of samples available from published data was limited, so we further used the scRNA-Seq data of annotated immune cells to generate 100 simulated bulk RNA-seq samples (further details are given in the *Materials and Methods* section). The TICPE showed a high correlation between the known proportions and the estimated fractions in the simulated dataset (*P <*0.01; **Figure 2D**). Furthermore, we used two scRNA-Seq datasets from melanoma patients (GSE115978; GSE72056) as benchmark resources for assessing the performance of the TICPE. The estimated proportion of each immune cell type was found to correlate with the true immune cell proportions that were calculated from single cell barcode information (*P <*0.05; **Supplementary Figures 1A, B**). Similarly, we used one of the scRNA-seq datasets (GSE115978) from melanoma ecosystems to simulate bulk samples of known cell type proportions and observed a good agreement with our predictions (*P <*0.05; **Supplementary Figure 1C**). Furthermore, we employed the receiver operating characteristic (ROC) curve and the area under the curve (AUC) to evaluate the performance of TICPE. We used the median true cell proportions as the cutoff for each cell type, and found the TICPE estimates had an AUC value ranging from 0.667 to 1 on publicly available datasets (**Supplementary Figure 2**).

**Figure 3:**
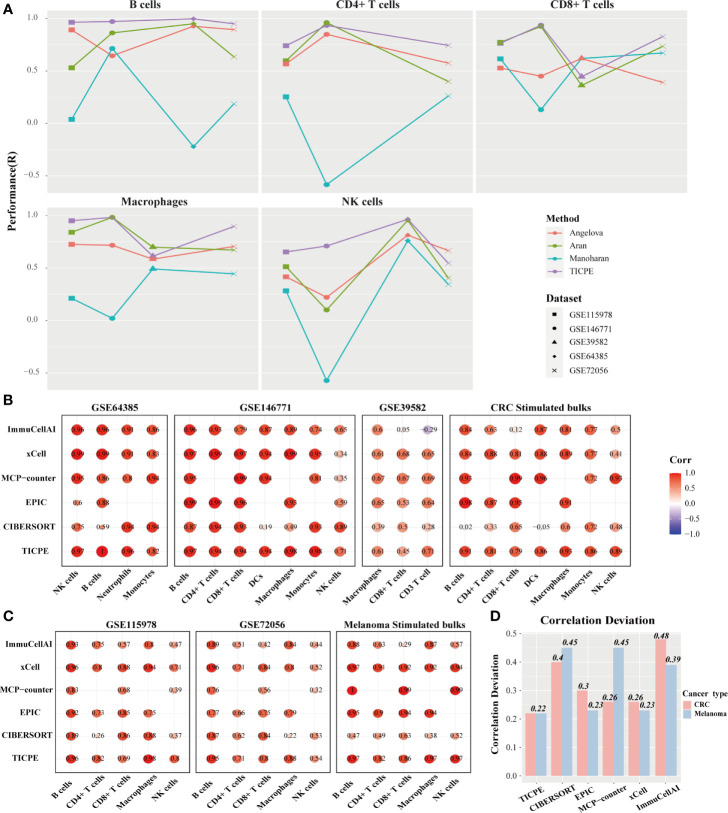
Performance comparison with other methods. **(A)** Scatter plot organized by cell type showing the performance of different marker gene sets identified from four sources. Methods performance was quantified using Pearson’s correlation (R). Different colors represented marker genes collated from different methods and different datasets had corresponding shapes. **(B, C)** Performance of TICPE and other methods on CRC and melanoma validation cohorts, respectively. Here rows corresponded to methods and columns showed the Pearson correlation coefficient for the corresponding cell type in each dataset. **(D)** Correlation deviation of each method in both CRC and melanoma validation datasets.

We hypothesized that the high correlation values resulted, at least in part, from the robust signature genes for a specific type of cancer. Therefore, we respectively collated sets of marker genes, as reported by Angelova et al. (30), Aran et al. (18), and Manoharan et al. (31), and used the same pipeline to estimate the cell proportions to compare the representativeness of the signature genes selected by the TICPE and those in specific types of cancer. Using these public validation datasets with different cell types, we evaluated the performance of the signature gene sets on independent datasets. We observed that, in most cases, the selected signature genes in our study showed better performance in terms of the estimated cell proportions than the marker genes from previously published methods (**Figure 3A**). In general, and not only for colorectal cancer and melanoma, the TICPE can also be developed to estimate the proportions of infiltrating immune cells in other types of cancer when collect relevant cancer cells and normal cells are tested within the same development pipeline. Moreover, the more robust cell type-specific genes we attained, the better performance we saw in the TICPE.

### A Comparison of the TICPE With Previously Published Methods

We utilized the public validation datasets and simulated RNA-seq data to benchmark a range of other methods (CIBERSORT, EPIC, MCP-counter, xCell, and ImmuCellAI) in order to predict immune cell proportions. Compared to the other methods used currently in CRC validation datasets, the TICPE did not obtain the highest correlations across all cell types; however, it did provide the most consistent performance of all the assessments (**Figure 3B**). The majority of cell types measured by TICPE showed higher correlations with the observed cell fractions than the other methods for both scRNA-Seq datasets from melanoma patients and simulated samples (**Figure 3C**). Performance of TICPE and previous computational methods was assessed with all validation datasets by cell type. We chose the cell type that was analyzed in more than three datasets. The TICPE robustly obtained positive correlations across all cell types and data sets and scored the high performers in the assessments (**Supplementary Figure 3A**). In addition, the TICPE showed the least correlation deviation for the publicly available datasets for CRC and melanoma (**Figure 3D**).

It is also worth noting that the available methods for estimating immune cell contents are based on quantitative expression measurements of reference profiles or signature genes, thus resulting in incomparability across different datasets. In contrast, our method was based on the relative ordering of gene expression and was developed to estimate cell proportions in every individual tumor sample; this strategy was more flexible than the other methods. We analyzed the cell infiltration of two scRNA-Seq datasets for melanoma and compared the results of *in silico* methods with the true proportions. With the exception of macrophages (Wilcoxon test; *P* = 0.017), we found that there was no significant difference in the actual cell fractions when compared between the two datasets for melanoma. The TICPE estimates, had a similar trend to the actual proportions and showed no statistical significance between the two datasets except for macrophages (Wilcoxon test; *P* = 0.02; **Supplementary Figure 3B**). However, the majority of cell contents estimated by xCell and ImmuCellAI between the two datasets were significantly different and differed from the actual proportions. These results showed that the TICPE is a robust approach that supports the comparisons of the same cell type across different datasets at the same time and shows high levels of accuracy and robustness to estimate the proportion of immune cells of tumor samples.

### TICPE Revealed That Immune Cell Infiltration Has Prognostic Value

**Figure 4:**
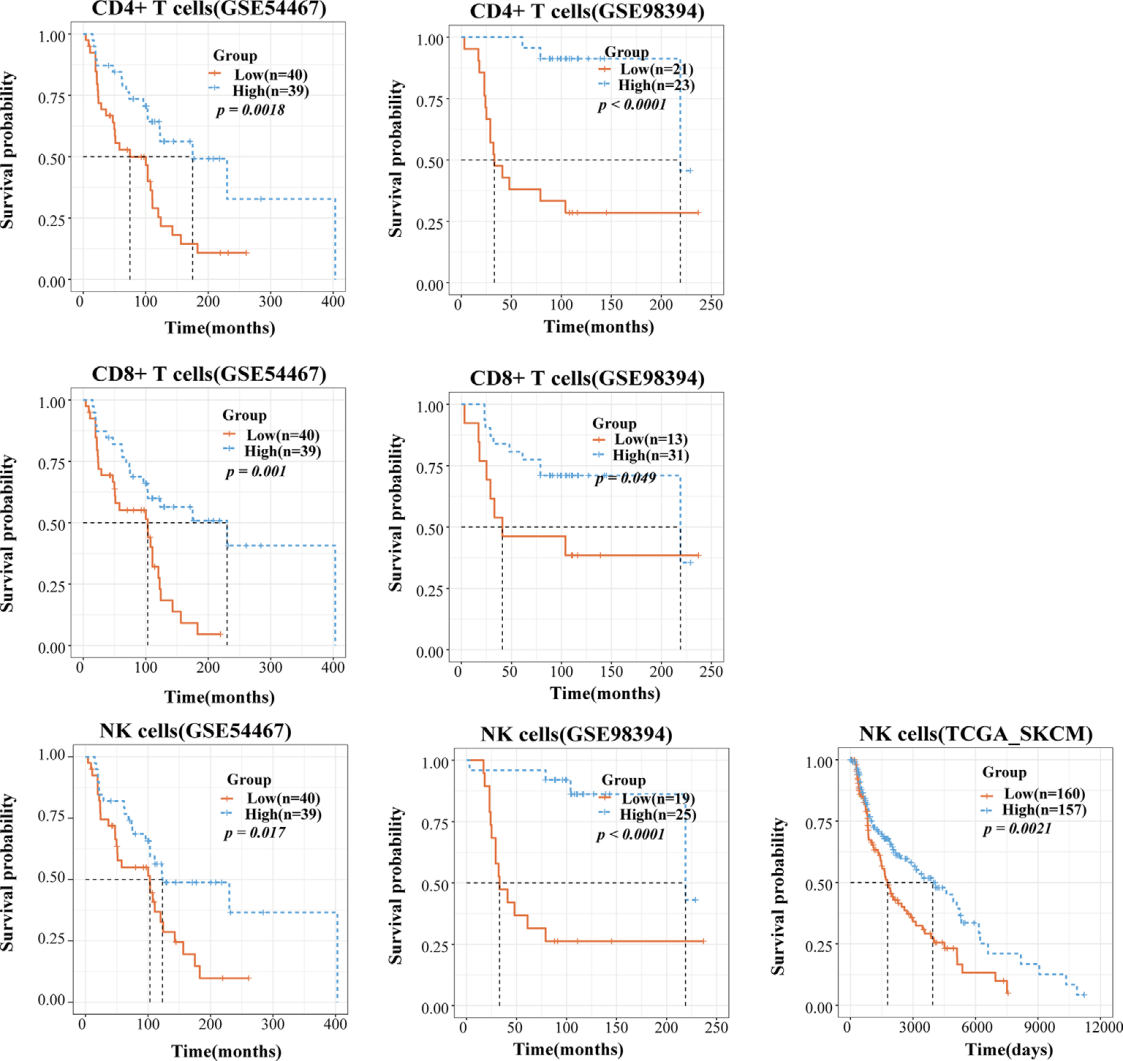
The application of TICPE on prognostic analysis for melanoma. Survival high CD4+ T/CD8+ T/NK cells and low CD4+ T/CD8+ T/NK cells groups in melanoma patients, respectively. *P* values comparing two groups were calculated with the log-rank test.

TIICs are indispensable components of the tumor microenvironment and have been demonstrated to be highly valuable in determining the prognosis of multiple cancers. We accessed data from the GEO and TCGA to investigate whether TIICs had prognosis value for melanoma patients. We employed the TICPE to systematically estimate the eight infiltrated immune cells and stratified patients into a high infiltration subtype and a low infiltration subtype by using the median cell proportions as the cutoff. The results of Kaplan–Meier analysis of 79 metastatic melanoma specimens (GSE54467) and a unique set of 51 treatment-naive primary melanoma samples (GSE98394) both indicated that higher fractions of CD4+ T, CD8+ T cells, and NK cells might be associated with better survival over those with low proportions (*P* <0.05; **Figure 4**). In addition, RNA-seq data from 472 SKCM patients and OS data for 468 patients were downloaded from the TCGA database. In addition, 323 patients with a blank therapy type were posited without chemo/radiotherapy. We only selected these patients to reduce the treatment affecting patient prognosis and also found that melanoma patients with a high density of NK cells had a better prognosis (*P* = 0.0021; **Figure 4**). Furthermore, the TICPE was able to estimate cell proportions in every individual tumor sample. Therefore, we combined 57 melanoma patients with lymphnode (GSE22153) and subcutaneous metastases, along with 20 melanoma patients with liver and lymphnode metastases (GSE22154), who were treated in the same clinical center. Melanoma patients with a higher abundance of B cells, CD4+ T, and CD8+ T cells had a longer overall survival with or without combining the 20 patients with liver and lymphnode metastases (**Supplementary Figure 4**).

We also applied the TICPE to four cohorts of CRC patients to investigate the relationship between cell infiltrations and patient prognosis. In a cohort of 160 stage II and III CRC tissue samples that were treated surgically (GSE24551), patients with high levels of CD8+ T cell infiltration were significantly associated with a better DFS (disease-free survival) compared with those with a low infiltration subtype (*P* = 0.041). In a large series of CRC patients who had not received adjuvant chemotherapy (GSE39582), and a cohort comprising 232 colorectal cancer patients (GSE17538), we found that a higher proportion of NK cells indicated a prolonged period of patient survival (*P* = 0.0079; *P* = 0.018) while an increased number of CD8+ T cells was associated with a better prognosis, although this was not statistically significant in either of the datasets. Furthermore, in a cohort of 232 colorectal cancer patients (GSE17538), despite the fact that macrophage infiltration was not statistically significant, a higher proportion of macrophages was associated with a dismal prognosis (*P* = 0.17). We also observed the same tendency in 171 surgically resected CRC specimens without chemo/radiotherapy (GSE14333); relatively poor DFS was correlated with an increased fraction of macrophages (*P* = 0.06; **Supplementary Figure 5**). Taken together, CD8+ T cells and NK cells were shown to play favorable roles in the survival of CRC and melanoma patients and the data obtained using the TICPE for several cohorts suggested that immune cell proportions can serve as an effective prognostic indicator for tumors.

### TICPE Detected Changes in the Proportions of Immune Cells During Immunotherapy for Melanoma

**Figure 5:**
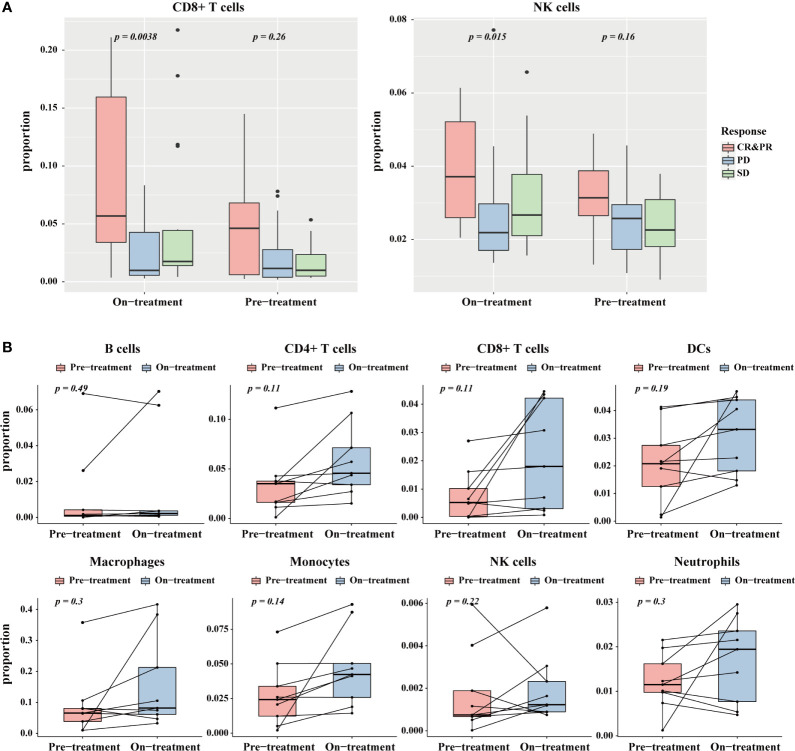
The application of TICPE on immunotherapy for melanoma. **(A)** The significant proportion differences of CD8+ T cells (left)/NK cells (right) in different response groups at pre- and on-treatment (anti-PD1) time point. **(B)** Change of the estimated immune cell proportions between pre-treatment and on-treatment time point in paired responders.

An increasing number of research studies has revealed that an elevation in the levels of CD8+ T cells and NK cells is associated with an immunotherapy response in anti-PD-1 treatment (32, 33). We applied the TICPE to a melanoma dataset (GSE91061) to investigate the impact of immune cell proportions on cancer immunotherapy. The estimated fractions of CD8+ T cells and NK cells in pre-treatment and on-treatment samples showed a substantial increment in the complete and partial response group (CR & PR) (Kruskal-Wallis test; *P* < 0.05) than the other groups (**Figure 5A**). With regards to pre-treatment data, the immune cell fractions across different groups showed no statistical differences. Notably, there was no statistically significant difference between paired pre-treatment *versus* on-treatment immune cell proportions in responders. However, there was an increasing trend for changes in immune cell proportions during anti-PD-1 treatment; this indicated that these immune cells were associated with a favorable response to PD-1 inhibition (**Figure 5B**; Wilcoxon test). These results suggested that TICPE could provide important insights on the dynamic immune cell infiltration during immunotherapy and offer valuable indicators for immunotherapy response during treatment.

## Discussion

In this study, we developed the TICPE, a cancer-specific qualitative method based on REOs, to estimate the proportion of eight different immune cells. The results of our extensive validation using immunohistochemistry data, mRNA mixtures *in vitro*, scRNA-Seq data, and simulated bulk RNA-seq samples, demonstrated that the TICPE could effectively infer immune cell fractions from transcriptome profiles. Of note, the TICPE does not only apply for colorectal cancer and melanoma, the TICPE could also be developed to estimate the infiltrating immune cells in any type of cancer as long as relevant data is available, such as cancer cells and normal samples. We had a straightforward comparison tumor microenvironment between colorectal cancer and melanoma utilizing data compiled by TCGA. The results showed that melanoma was highly infiltrated by CD4+ T cells, NK cells, dendritic cells, and neutrophils but poorly by cells of B cells, CD8+ T cells, monocytes, and macrophages in comparison with colorectal cancer (**Supplementary Figure 6A**). Moreover, the TICPE could be broadly employed to other components of the tumor microenvironment with the increased availability of public data by using the proposed pipeline. In the further work, with the gradual accumulation of relevant data of other cancer types and cell types, we will develop the TICPE for each neoplasm. Then we will estimate the abundance of immune cell populations in samples across multiple cancer types from The Cancer Genome Atlas (TCGA), and propose a global analysis of immune landscape across human cancers.

The key step in constructing the TICPE involved accurately identifying a list of genes characterized by a cell type. Compared to other methods based on marker genes, our method was more reliable due to the fact that it incorporated a group of signature genes for each cell type that was acquired from a comprehensive literature search and featured differentially expressed genes when compared with cancer cells. As shown the Venn diagram of **Supplementary Figure 6B**, there were some shared cell type-specific genes in both CRC and melanoma, but the majority of signature genes for each cell type were cancer type-specific in our study (**Supplementary Table 4**), which also indicated the gene expression of immune cell varied across different tissues. On the other hand, we chose to apply a gene signature approach over deconvolution methods because of the several advantages that the former provides. First, we did not require a reference expression matrix and could treat immune cells independently; this supported inter-sample comparisons and avoided issues relating to multicollinearity. Second, the TICPE was based on the rank of gene expression rather than the actual gene expression value and was therefore suitable for cross-platform transcriptomic measurements and comparisons. Finally, gene signatures are simple and can easily be adjusted. Furthermore, the procedure for developing TICPE was based on REOs so that it was agnostic to monotonic data normalization or concerns related to experimental batch effects; these effects rendered our technique more robust to both technical and biological noise.

The TICPE was reliable and could stratify a cohort of similar tumors based on the composition of their immune microenvironments, and could follow proportional changes of the microenvironment during the course of immunotherapy. In this investigation, CD8+ T cells and NK cells were shown to play favorable roles in the survival of CRC and melanoma patients. CD8+ T cells are the most potent cytolytic cell subset and NK cells also exert cytolytic functions (34, 35). CD8+ T cells are able to exert a directly killing effect on tumors cells and have been linked to a better prognosis in several types of cancer (36). In parallel with CD8+ T cells, NK cells can recognize and kill neoplastic cells and play pivotal roles in innate and adaptive immune responses and tumor immunosurveillance (37). In addition, our study also showed that the abundance of macrophages may serve as an unfavorable prognostic marker for CRC. Macrophages are conventionally classified into M1 and M2 subtypes. M2 macrophages secrete Interleukin 10 (IL-10), transforming growth factor-β(TGF-β), and other mediators that stimulate tumor-related angiogenesis and inhibit antitumor immune response (4, 38). On the other hand, although limited in sample size, our analysis detected an association between anti-PD-1 immunotherapy response and elevated CD8+ T cell and NK cell levels and revealed the potential of TICPE for providing important insights into dynamic immune cell infiltration during immunotherapy. The TIICs found in our study make a significant contribution to patient survival and treatment and our findings are corroborated by previous studies (17, 39). Overall, the proportions of immune cells measured by the TICPE could serve as a prognostic factor or a potential predictive model for the response to immune checkpoint blockade therapy in solid tumors.

Despite the utility of our method for estimating the tumor immune contexture for a particular type of cancer, several issues require further investigation. First, the TICPE was a cancer specific model and only focused on a very narrow range of cell types from the tumor microenvironment as our research involved publicly available datasets and gene sets that were characterized by each type of immune cell. Further efforts are required to collect more relevant information to extend the technique to other cell types (e.g., cancer-associated fibroblasts) and should be expanded to include more cancer types. Moreover, the inferences were strictly upregulated scores which could be compared with cancer cell and could not be interpreted as proportions. Thus, while we attempted to calibrate our method to resemble proportions, this strategy was hindered by conducting simulations to real-world datasets; the reliability of the technique needs to be improved. Furthermore, the final estimates were not normalized to sum up to one; therefore, the estimates could not be interpreted directly as cell fractions. Consequently, further improvement of our method is certainly warranted, including the reselection of signature genes from genome-wide genes to extend to other cell types and the selection of a large cohort of real-world datasets with cell proportions to conduct simulated models.

In summary, the TICPE is a cancer-specific qualitative method for estimating the tumor immune contexture using public RNA-Seq and microarray datasets. The TICPE can estimate the proportion of infiltrating immune cells in CRC and melanoma but could also be extended to other types of cancer. Furthermore, the TICPE was based on REOs and can estimate the proportions of tumor-infiltrating immune cells in individual tumor samples. Therefore, the TICPE showed good comparability across different datasets and was only weakly affected by batch effects. We anticipate that this method will assist in the discovery of novel prognostic and predictive response biomarkers for both conventional and immunotherapy by taking immune cell composition into account.

## Data Availability Statement

The datasets presented in this study can be found in online repositories. The names of the repository/repositories and accession number(s) can be found in the article/**Supplementary Material**.

## Author Contributions

WZ and YH conceived of, designed, and supervised the study. HX, JZ, and KW drafted the manuscript. HX and JZ implemented the source code of TICPE. KW participated in the study design and result validation. KS, HZ, JY, KL, and RY contributed to the design of TICPE. All authors contributed to the article and approved the submitted version.

## Funding

This work was supported by the National Natural Science Foundation of China under Grant: 81872396.

## Conflict of Interest

The authors declare that the research was conducted in the absence of any commercial or financial relationships that could be construed as a potential conflict of interest.

## References

[1] Petitprez F Meylan M de Reynies A Sautes-Fridman C Fridman WH. The Tumor Microenvironment in the Response to Immune Checkpoint Blockade Therapies. Front Immunol (2020) 11:784. 10.3389/fimmu.2020.00784 32457745 PMC7221158

[2] Galon J Costes A Sanchez-Cabo F Kirilovsky A Mlecnik B Lagorce-Pages C. Type, Density, and Location of Immune Cells Within Human Colorectal Tumors Predict Clinical Outcome. Science (2006) 313(5795):1960–4. 10.1126/science.1129139 17008531

[3] Sideras K Galjart B Vasaturo A Pedroza-Gonzalez A Biermann K Mancham S. Prognostic Value of Intra-Tumoral CD8(+) /FoxP3(+) Lymphocyte Ratio in Patients With Resected Colorectal Cancer Liver Metastasis. J Surg Oncol (2018) 118(1):68–76. 10.1002/jso.25091 29878369 PMC6175125

[4] Lan J Sun L Xu F Liu L Hu F Song D. M2 Macrophage-Derived Exosomes Promote Cell Migration and Invasion in Colon Cancer. Cancer Res (2019) 79(1):146–58. 10.1158/0008-5472.CAN-18-0014 30401711

[5] Nishikawa H Sakaguchi S. Regulatory T Cells in Cancer Immunotherapy. Curr Opin Immunol (2014) 27:1–7. 10.1016/j.coi.2013.12.005 24413387

[6] Maibach F Sadozai H Seyed Jafari SM Hunger RE Schenk M. Tumor-Infiltrating Lymphocytes and Their Prognostic Value in Cutaneous Melanoma. Front Immunol (2020) 11:2105. 10.3389/fimmu.2020.02105 33013886 PMC7511547

[7] Subrahmanyam PB Dong Z Gusenleitner D Giobbie-Hurder A Severgnini M Zhou J. Distinct Predictive Biomarker Candidates for Response to anti-CTLA-4 and anti-PD-1 Immunotherapy in Melanoma Patients. J Immunother Cancer (2018) 6(1):18. 10.1186/s40425-018-0328-8 29510697 PMC5840795

[8] Tietze JK Angelova D Heppt MV Reinholz M Murphy WJ Spannagl M. The Proportion of Circulating CD45RO(+)CD8(+) Memory T Cells is Correlated With Clinical Response in Melanoma Patients Treated With Ipilimumab. Eur J Cancer (2017) 75:268–79. 10.1016/j.ejca.2016.12.031 28242504

[9] Fridman WH Zitvogel L Sautes-Fridman C Kroemer G. The Immune Contexture in Cancer Prognosis and Treatment. Nat Rev Clin Oncol (2017) 14(12):717–34. 10.1038/nrclinonc.2017.101 28741618

[10] Chen Z Wu A. Progress and Challenge for Computational Quantification of Tissue Immune Cells. Brief Bioinform (2021) bbaa358. 10.1093/bib/bbaa358 33401306

[11] Finotello F Trajanoski Z. Quantifying Tumor-Infiltrating Immune Cells From Transcriptomics Data. Cancer Immunol Immunother (2018) 67(7):1031–40. 10.1007/s00262-018-2150-z PMC6006237 29541787

[12] Sturm G Finotello F Petitprez F Zhang JD Baumbach J Fridman WH. Comprehensive Evaluation of Transcriptome-Based Cell-Type Quantification Methods for Immuno-Oncology. Bioinformatics (2019) 35(14):i436–45. 10.1093/bioinformatics/btz363 PMC6612828 31510660

[13] Newman AM Liu CL Green MR Gentles AJ Feng W Xu Y. Robust Enumeration of Cell Subsets From Tissue Expression Profiles. Nat Methods (2015) 12(5):453–7. 10.1038/nmeth.3337 PMC4739640 25822800

[14] Li B Severson E Pignon JC Zhao H Li T Novak J. Comprehensive Analyses of Tumor Immunity: Implications for Cancer Immunotherapy. Genome Biol (2016) 17(1):174. 10.1186/s13059-016-1028-7 27549193 PMC4993001

[15] Racle J de Jonge K Baumgaertner P Speiser DE Gfeller D. Simultaneous Enumeration of Cancer and Immune Cell Types From Bulk Tumor Gene Expression Data. Elife (2017) 6:e26476. 10.7554/eLife.26476 29130882 PMC5718706

[16] Finotello F Mayer C Plattner C Laschober G Rieder D Hackl H. Molecular and Pharmacological Modulators of the Tumor Immune Contexture Revealed by Deconvolution of RNA-seq Data. Genome Med (2019) 11(1):34. 10.1186/s13073-019-0638-6 31126321 PMC6534875

[17] Becht E Giraldo NA Lacroix L Buttard B Elarouci N Petitprez F. Estimating the Population Abundance of Tissue-Infiltrating Immune and Stromal Cell Populations Using Gene Expression. Genome Biol (2016) 17(1):218. 10.1186/s13059-016-1070-5 27765066 PMC5073889

[18] Aran D Hu Z Butte AJ. xCell: Digitally Portraying the Tissue Cellular Heterogeneity Landscape. Genome Biol (2017) 18(1):220. 10.1186/s13059-017-1349-1 29141660 PMC5688663

[19] Miao YR Zhang Q Lei Q Luo M Xie GY Wang H. Immucellai: A Unique Method for Comprehensive T-Cell Subsets Abundance Prediction and its Application in Cancer Immunotherapy. Adv Sci (Weinh) (2020) 7(7):1902880. 10.1002/advs.201902880 32274301 PMC7141005

[20] Frishberg A Steuerman Y Gat-Viks I. CoD: Inferring Immune-Cell Quantities Related to Disease States. Bioinformatics (2015) 31(24):3961–9. 10.1093/bioinformatics/btv498 26315914

[21] Lazar C Meganck S Taminau J Steenhoff D Coletta A Molter C. Batch Effect Removal Methods for Microarray Gene Expression Data Integration: A Survey. Brief Bioinform (2013) 14(4):469–90. 10.1093/bib/bbs037 22851511

[22] Patil P Bachant-Winner PO Haibe-Kains B Leek JT. Test Set Bias Affects Reproducibility of Gene Signatures. Bioinformatics (2015) 31(14):2318–23. 10.1093/bioinformatics/btv157 PMC4495301 25788628

[23] Qi L Li T Shi G Wang J Li X Zhang S. An Individualized Gene Expression Signature for Prediction of Lung Adenocarcinoma Metastases. Mol Oncol (2017) 11(11):1630–45. 10.1002/1878-0261.12137 PMC5663997 28922552

[24] Eisenhauer EA Therasse P Bogaerts J Schwartz LH Sargent D Ford R. New Response Evaluation Criteria in Solid Tumours: Revised RECIST Guideline (Version 1.1). Eur J Cancer (2009) 45(2):228–47. 10.1016/j.ejca.2008.10.026 19097774

[25] Wang H Sun Q Zhao W Qi L Gu Y Li P. Individual-Level Analysis of Differential Expression of Genes and Pathways for Personalized Medicine. Bioinformatics (2015) 31(1):62–8. 10.1093/bioinformatics/btu522 25165092

[26] Korthauer K Kimes PK Duvallet C Reyes A Subramanian A Teng M. A Practical Guide to Methods Controlling False Discoveries in Computational Biology. Genome Biol (2019) 20(1):118. 10.1186/s13059-019-1716-1 31164141 PMC6547503

[27] Johnson WE Li C Rabinovic A. Adjusting Batch Effects in Microarray Expression Data Using Empirical Bayes Methods. Biostatistics (2007) 8(1):118–27. 10.1093/biostatistics/kxj037 16632515

[28] Aran D Sirota M Butte AJ. Systematic Pan-Cancer Analysis of Tumour Purity. Nat Commun (2015) 6:8971. 10.1038/ncomms9971 26634437 PMC4671203

[29] Bland JM Altman DG. The Logrank Test. BMJ (2004) 328(7447):1073. 10.1136/bmj.328.7447.1073 15117797 PMC403858

[30] Angelova M Charoentong P Hackl H Fischer ML Snajder R Krogsdam AM. Characterization of the Immunophenotypes and Antigenomes of Colorectal Cancers Reveals Distinct Tumor Escape Mechanisms and Novel Targets for Immunotherapy. Genome Biol (2015) 16:64. 10.1186/s13059-015-0620-6 25853550 PMC4377852

[31] Manoharan M Mandloi N Priyadarshini S Patil A Gupta R Iyer L. A Computational Approach Identifies Immunogenic Features of Prognosis in Human Cancers. Front Immunol (2018) 9:3017. 10.3389/fimmu.2018.03017 30622534 PMC6308325

[32] Eroglu Z Zaretsky JM Hu-Lieskovan S Kim DW Algazi A Johnson DB. High Response Rate to PD-1 Blockade in Desmoplastic Melanomas. Nature (2018) 553(7688):347–50. 10.1038/nature25187 PMC5773412 29320474

[33] Iraolagoitia XL Spallanzani RG Torres NI Araya RE Ziblat A Domaica CI. NK Cells Restrain Spontaneous Antitumor CD8+ T Cell Priming Through PD-1/PD-L1 Interactions With Dendritic Cells. J Immunol (2016) 197(3):953–61. 10.4049/jimmunol.1502291 27342842

[34] de Wolf C van de Bovenkamp M Hoefnagel M. Regulatory Perspective on In Vitro Potency Assays for Human T Cells Used in Anti-Tumor Immunotherapy. Cytotherapy (2018) 20(5):601–22. 10.1016/j.jcyt.2018.01.011 29598903

[35] Guo L Wang C Qiu X Pu X Chang P. Colorectal Cancer Immune Infiltrates: Significance in Patient Prognosis and Immunotherapeutic Efficacy. Front Immunol (2020) 11:1052. 10.3389/fimmu.2020.01052 32547556 PMC7270196

[36] Oh S Perera LP Terabe M Ni L Waldmann TA Berzofsky JA. IL-15 as a Mediator of CD4+ Help for CD8+ T Cell Longevity and Avoidance of TRAIL-mediated Apoptosis. Proc Natl Acad Sci USA (2008) 105(13):5201–6. 10.1073/pnas.0801003105 PMC2278231 18362335

[37] Bruni D Angell HK Galon J. The Immune Contexture and Immunoscore in Cancer Prognosis and Therapeutic Efficacy. Nat Rev Cancer (2020) 20(11):662–80. 10.1038/s41568-020-0285-7 32753728

[38] Fridman WH Pages F Sautes-Fridman C Galon J. The Immune Contexture in Human Tumours: Impact on Clinical Outcome. Nat Rev Cancer (2012) 12(4):298–306. 10.1038/nrc3245 22419253

[39] Tang D Park S Zhao H. NITUMID: Nonnegative Matrix Factorization-Based Immune-TUmor Microenvironment Deconvolution. Bioinformatics (2020) 36(5):1344–50. 10.1093/bioinformatics/btz748 PMC8215918 31593244

