# Supplementary material for: A Cancer-Specific Qualitative Method for Estimating the Proportion of Tumor-Infiltrating Immune Cells
Source: Front Immunol. 2021 May 14;12:672031. doi: 10.3389/fimmu.2021.672031 (PMC8160514; doi:10.3389/fimmu.2021.672031)
Supplement: Supplementary file 1 [file DataSheet_1.docx]

***Supplementary Material***

# Supplementary Figures and Tables

## Supplementary Figures

**
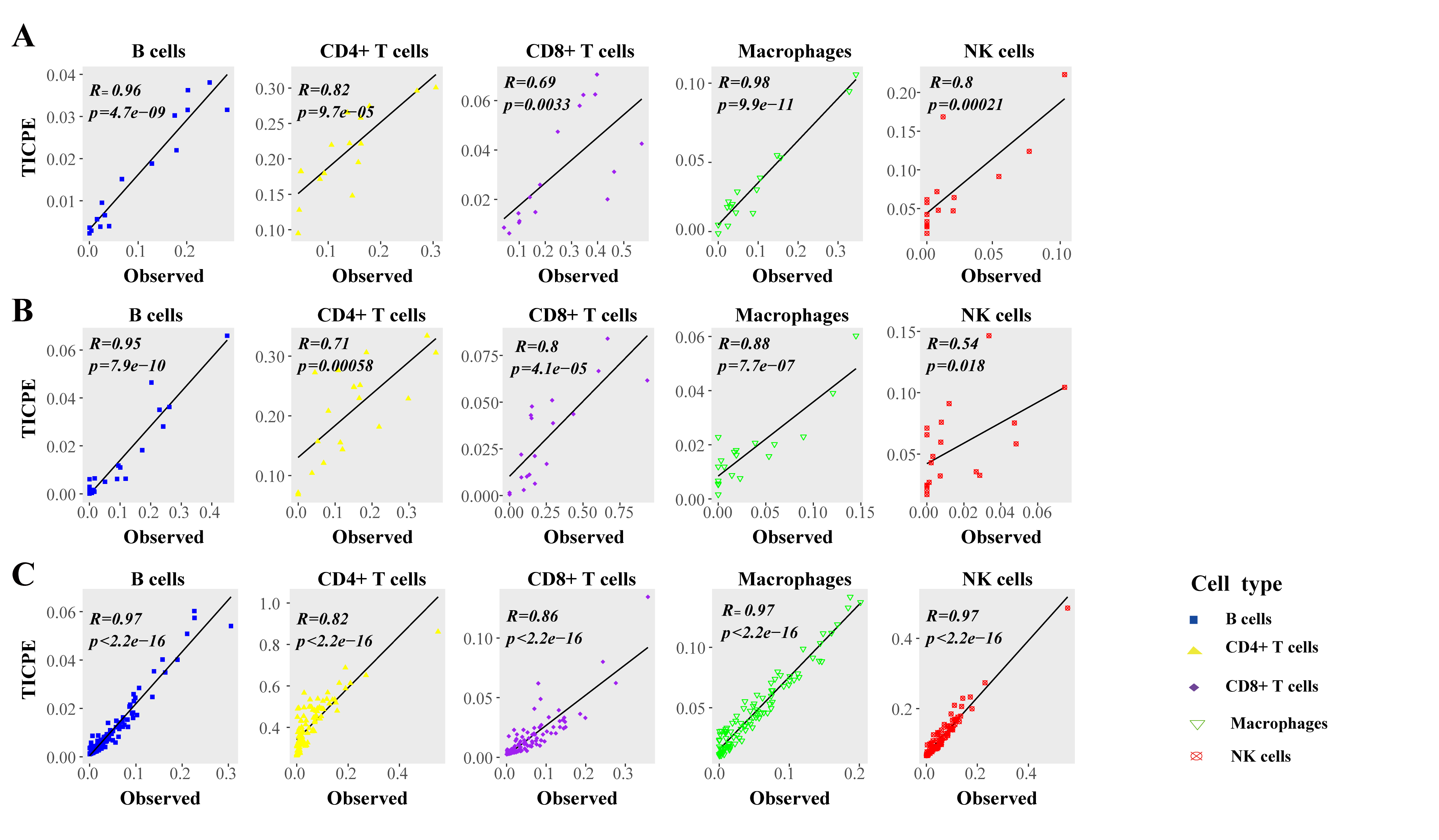
**

**Supplementary Figure 1. Performance assessment of the TICPE in melanoma. (A)** Comparison with scRNA-Seq data from GSE115978. **(B)** Comparison with scRNA-Seq data from GSE72056. **(C)** Comparison with known cell type fractions on 100 simulated bulk samples generated from scRNA-seq from melanoma samples.

**
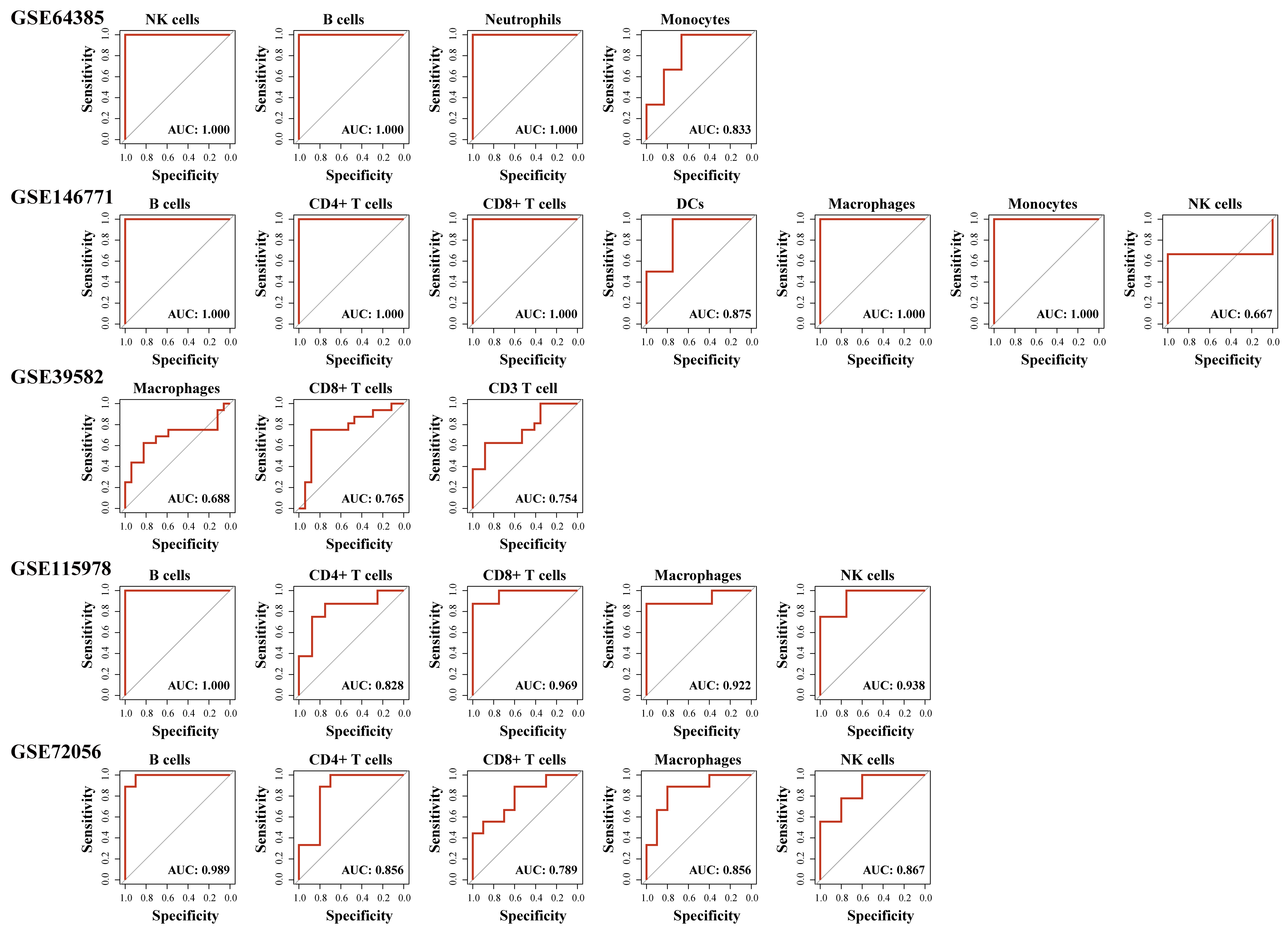
Supplementary Figure 2. AUC for the TICPE estimates on each publicly available data.**

**
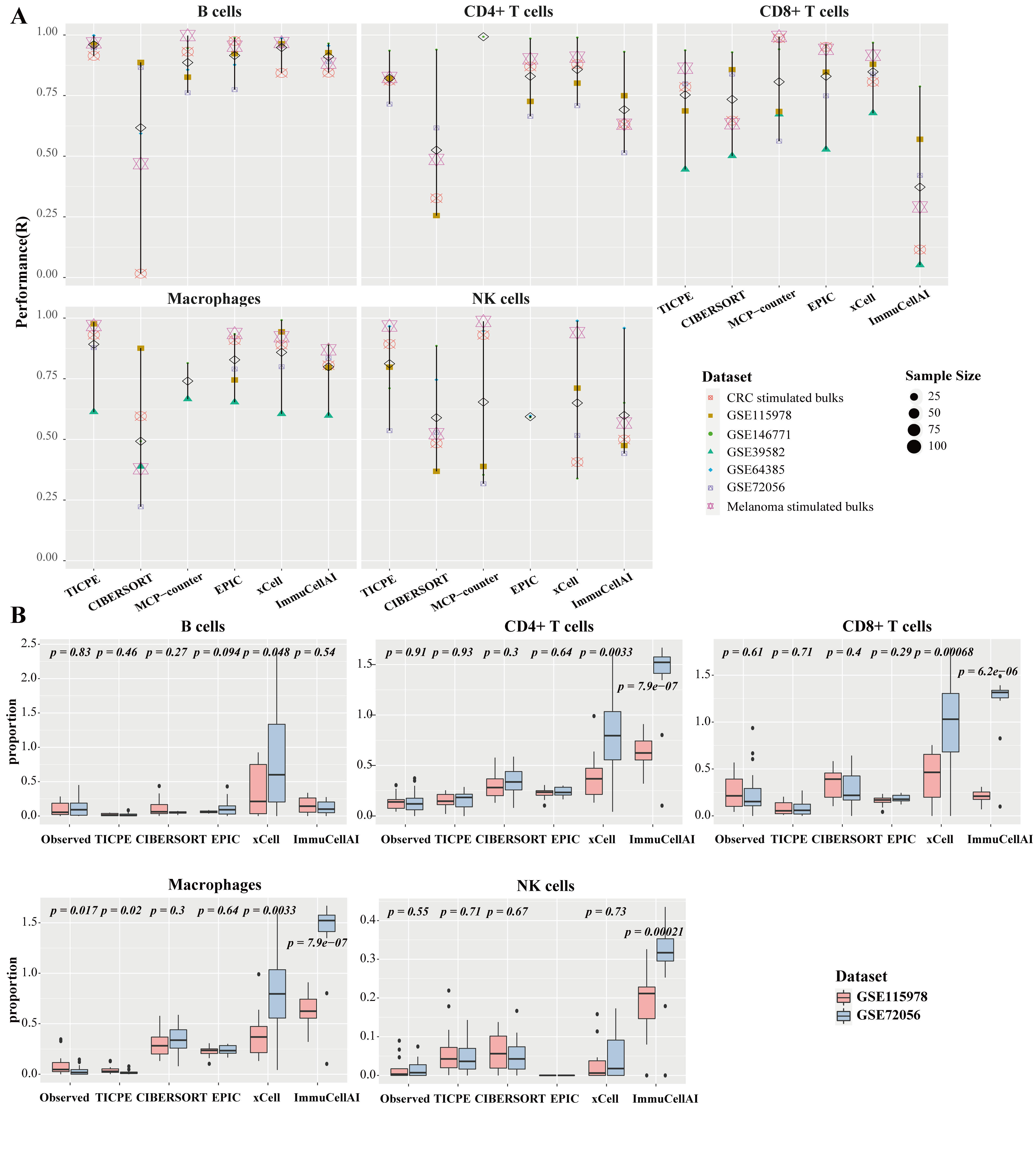
Supplementary Figure 3. A comparison of the TICPE with previously published methods. (A)** Performance of TICPE and previous computational methods was assessed with all validation datasets by cell type. **(B)** The significant difference of the estimated proportion of five types of immune cells by different methods between GSE115978 and GSE72056. Performance was Pearson’s correlation (R); Observed was the true immune cell proportions calculated from single cell barcode information.

**
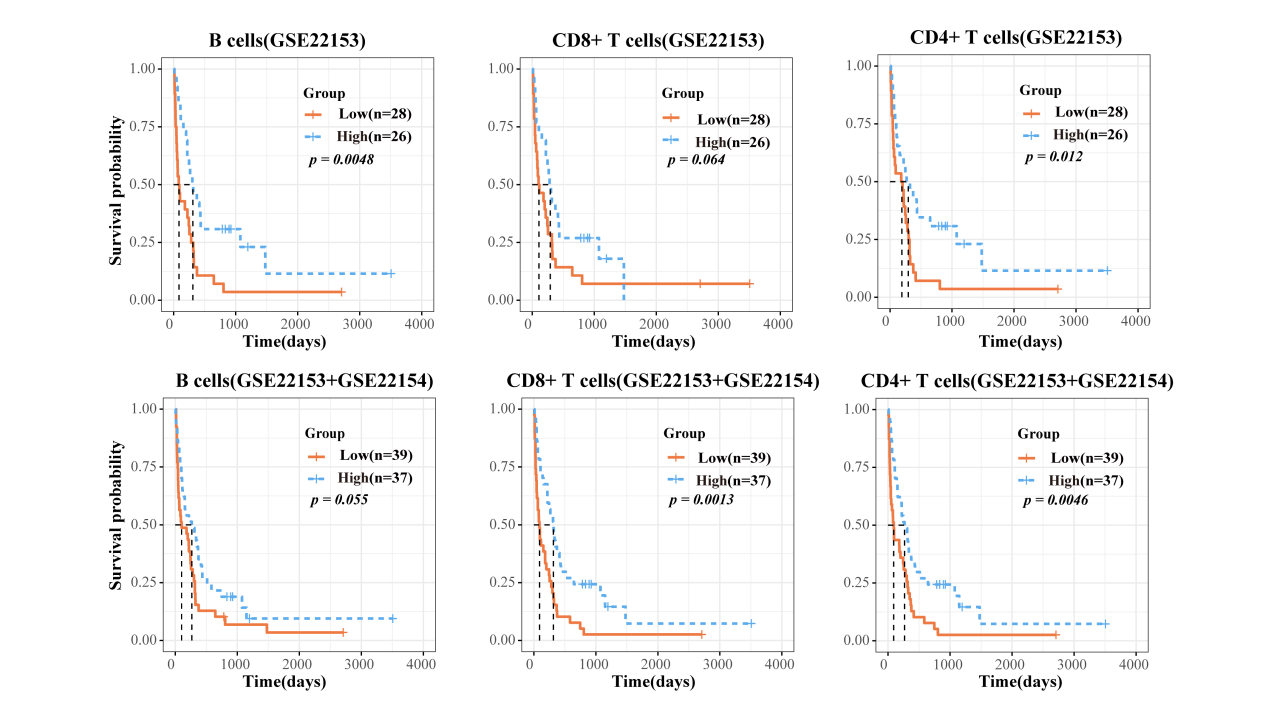
Supplementary Figure 4. The application of TICPE on prognostic analysis for melanoma.** Survival between B/CD4+ T/CD8+ T cells and low B/CD4+ T/CD8+ T cells groups in GSE22153 or in GSE22153 combined GSE22154.

**
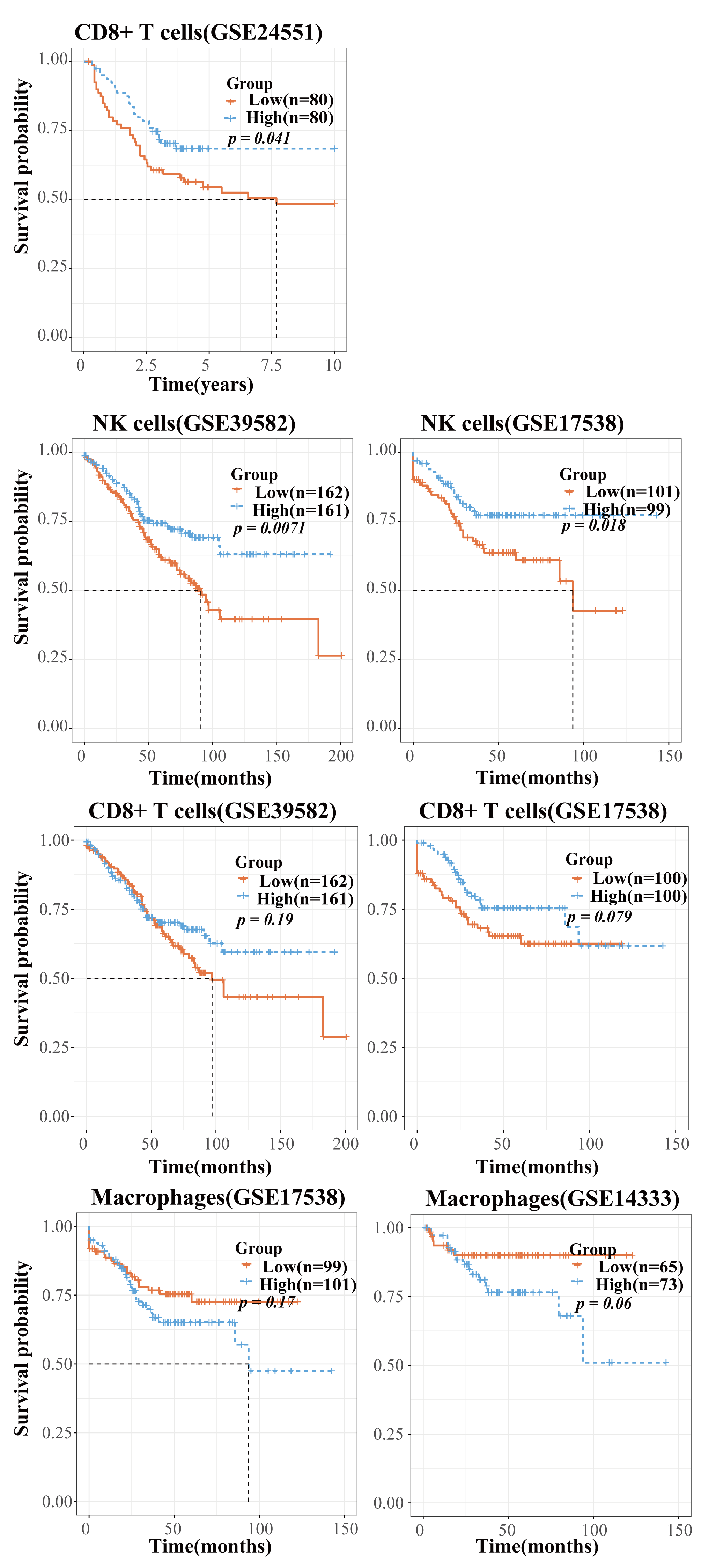
**

**Supplementary Figure 5. The application of TICPE on prognostic analysis for CRC.** Survival between high CD8+ T cells/NK cells/Macrophages and low CD8+ T cells/NK cells/Macrophages groups in CRC patients, respectively. P values comparing two groups were calculated with the log-rank test.

**
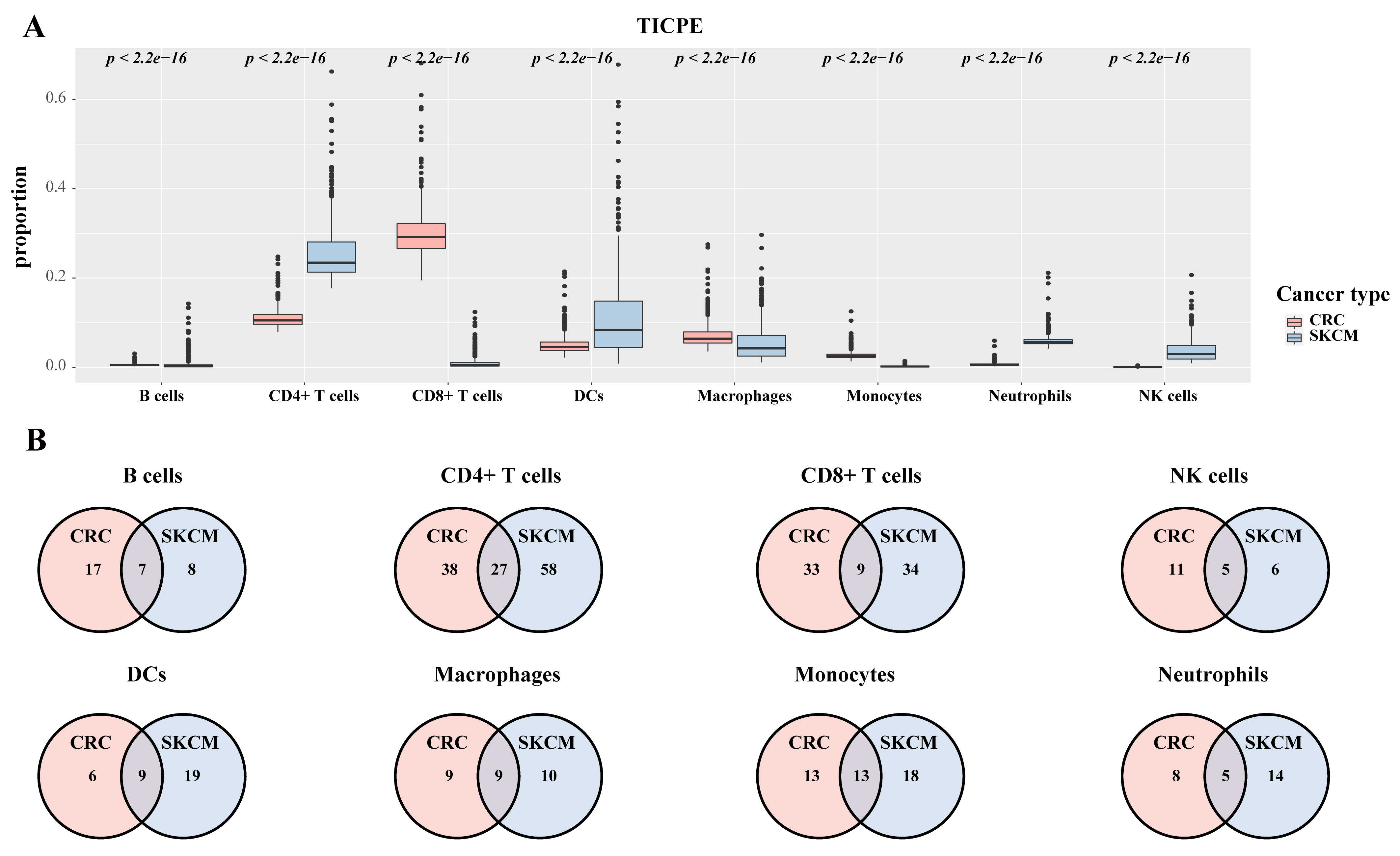
Supplementary Figure 6. The difference between colorectal cancer and melanoma.** (A) Comparison of the proportions of different infiltrating immune cells across colorectal cancer and melanoma tumors from TCGA. (B) Intersection signature genes of CRC and SKCM. Note: pink represents the number of specific signature genes for CRC, blue represents the number of specific signature genes for SKCM, and the middle part represents the intersection signature genes of the two. CRC: colorectal cancer; SKCM:melanoma.

## Supplementary Tables

**Supplementary Table1. Description of datasets used in this study.**

| **GEO ID** | **Platform** | **Data Type** | **Sample Size** | **Description** |
| --- | --- | --- | --- | --- |
| GSE11618 | Affymetrix GPL570 | HCT116 CRC cell lines | 18 | Generating signature genes &Development TICPE |
| GSE13059 | Affymetrix GPL570 | HT29 CRC cell lines | 23 |  |
| GSE14103 | Affymetrix GPL570 | HCT116 CRC cell lines | 12 |  |
| GSE16648 | Affymetrix GPL570 | Caco2、HT29 CRC cell lines | 12 |  |
| GSE18560 | Affymetrix GPL570 | Ls174T CRC cell lines | 12 |  |
| GSE24795 | Affymetrix GPL570 | 30 types of CRC cell lines | 30 |  |
| GSE35566 | Affymetrix GPL570 | 10 types of CRC cell lines | 19 |  |
| GSE55624 | Affymetrix GPL570 | SW480 CRC cell lines | 18 |  |
| GSE59196 | Affymetrix GPL570 | HCT15、HT115 CRC cell lines | 24 |  |
| GSE63252 | Affymetrix GPL570 | HCT116 CRC cell lines | 27 |  |
| GSE112282 | Affymetrix GPL570 | RKO、COLO 201 CRC cell lines | 24 |  |
| GSE116528 | Affymetrix GPL570 | 12 types of CRC cell lines | 12 |  |
| GSE90085 | Illumina GPL10558 | HCT116 CRC cell lines | 12 |  |
| GSE59883 | Illumina GPL10558 | NCIH508、HT29、SW403 CRC cell lines | 28 |  |
| GSE59857 | Illumina GPL10558 | 155 types of colorectal cancer cell lines | 155 |  |
| GSE116529 | Illumina GPL10558 | CaCo2、HT55、HCT116、RKO CRC cell lines | 24 |  |
| GSE75205 | Illumina GPL10558 | 6 types of CRC cell lines | 24 |  |
| GSE106073 | Illumina GPL10558 | LS174T CRC cell lines | 12 |  |
| GSE120993 | Illumina GPL10558 | C6244R、COLO205 CRC cell lines | 36 |  |
| GSE72544 | Illumina GPL10558 | 16 types of CRC cell lines | 36 |  |
| GSE50791 | Illumina GPL10558 | Colo-205、HT29 CRC cell lines | 18 |  |
| GSE119197 | Illumina GPL10558 | DLD-1 CRC cell lines | 12 |  |
| GSE115716 | Illumina GPL10558 | LS174T CRC cell lines | 27 |  |
| GSE122985 | Illumina GPL10558 | HCT116 CRC cell lines | 24 |  |
| GSE110425 | Illumina GPL10558 | HCT-116 CRC cell lines | 12 |  |
| GSE50841 | Illumina GPL10558 | SW480 CRC cell lines | 24 |  |
| GSE78995 | Affymetrix GPL570 | 59 early passage melanoma cell lines | 59 |  |
| GSE31534 | Affymetrix GPL570 | A375 melanoma cells | 51 |  |
| GSE10916 | Affymetrix GPL570 | 50 human-melanoma derived cell lines | 50 |  |
| GSE7127 | Affymetrix GPL570 | 63 types of melanoma cell lines | 63 |  |
| GSE68453 | Illumina GPL10558 | 16 types of melanoma cell lines | 180 |  |
| GSE50474 | Illumina GPL10558 | 1205Lu melanoma cell lines | 42 |  |
| GSE38466 | Illumina GPL10558 | 1205Lu melanoma cell lines | 42 |  |
| GSE24736 | Affymetrix GPL570 | B cells | 16 |  |
| GSE19599 | Affymetrix GPL570 | B cells | 6 |  |
| GSE12366 | Affymetrix GPL570 | B cells | 6 |  |
| GSE49910 | Affymetrix GPL570 | B cells | 4 |  |
| GSE75007 | Illumina GPL10558 | B cells | 63 |  |
| GSE120367 | Illumina GPL10558 | B cells | 117 |  |
| GSE11292 | Affymetrix GPL570 | CD4+ T cells | 81 |  |
| GSE36769 | Affymetrix GPL570 | CD4+ T cells | 60 |  |
| GSE32959 | Affymetrix GPL570 | CD4+ T cells | 37 |  |
| GSE50175 | Affymetrix GPL570 | CD4+ T cells | 8 |  |
| GSE71956 | Illumina GPL10558 | CD4+ T cells | 25 |  |
| GSE50392 | Illumina GPL10558 | CD4+ T cells | 48 |  |
| GSE84251 | Affymetrix GPL570 | CD8+ T cells | 12 |  |
| GSE93683 | Affymetrix GPL570 | CD8+ T cells | 48 |  |
| GSE98640 | Affymetrix GPL570 | CD8+ T cells | 30 |  |
| GSE84331 | Affymetrix GPL570 | CD8+ T cells | 12 |  |
| GSE71956 | Illumina GPL10558 | CD8+ T cells | 24 |  |
| GSE27838 | Affymetrix GPL570 | NK cells | 32 |  |
| GSE8059 | Affymetrix GPL570 | NK cells | 4 |  |
| GSE21774 | Affymetrix GPL570 | NK cells | 9 |  |
| GSE35330 | Affymetrix GPL570 | NK cells | 24 |  |
| GSE75091 | Illumina GPL10558 | NK cells | 24 |  |
| GSE16385 | Affymetrix GPL570 | Macrophages | 36 |  |
| GSE13670 | Affymetrix GPL570 | Macrophages | 30 |  |
| GSE7568 | Affymetrix GPL570 | Macrophages | 25 |  |
| GSE24897 | Affymetrix GPL570 | Macrophages | 12 |  |
| GSE100129 | Illumina GPL10558 | Macrophages | 24 |  |
| GSE102117 | Illumina GPL10558 | Macrophages | 9 |  |
| GSE38351 | Affymetrix GPL570 | Monocytes | 41 |  |
| GSE39840 | Affymetrix GPL570 | Monocytes | 10 |  |
| GSE35683 | Affymetrix GPL570 | Monocytes | 30 |  |
| GSE6054 | Affymetrix GPL570 | Monocytes | 23 |  |
| GSE60199 | Illumina GPL10558 | Monocytes | 10 |  |
| GSE98480 | Illumina GPL10558 | Monocytes | 12 |  |
| GSE7509 | Affymetrix GPL570 | DCs | 20 |  |
| GSE10316 | Affymetrix GPL570 | DCs | 13 |  |
| GSE23618 | Affymetrix GPL570 | DCs | 12 |  |
| GSE23371 | Affymetrix GPL570 | DCs | 12 |  |
| GSE87494 | Illumina GPL10558 | DCs | 19 |  |
| GSE85305 | Illumina GPL10558 | DCs | 16 |  |
| GSE22103 | Affymetrix GPL570 | Neutrophils | 22 |  |
| GSE39889 | Affymetrix GPL570 | Neutrophils | 16 |  |
| GSE8668 | Affymetrix GPL570 | Neutrophils | 24 |  |
| GSE18810 | Affymetrix GPL570 | Neutrophils | 6 |  |
| GSE70044 | Illumina GPL10558 | Neutrophils | 114 |  |
| GSE7821 | Affymetrix GPL570 | the intestinal biopsies of 40 healthy human twins | 40 |  |
| GSE15605 | Affymetrix GPL570 | 16 normal skin samples | 16 |  |
| GSE49910 | Affymetrix GPL570 | eight human immune cell subset | 745 |  |
| GSE24795 | Affymetrix GPL570 | 30 types of CRC cell lines | 30 |  |
| GSE7127b | Affymetrix GPL570 | 63 types of melanoma cell lines | 63 |  |
| GSE64385 | Affymetrix GPL570 | Immune and HCT116 RNA mixtures | 12 | Validation |
| GSE39582 | Affymetrix GPL570 | | 33 |  |
| GSE146771 | Illumina GPL20301 | | 10 |  |
| GSE115978 | Illumina GPL18573 | | 31 |  |
| GSE72056 | Illumina GPL18573 | | 19 |  |
| GSE22153 | Illumina GPL6102 | 57 lymphnode and subcutaneous melanoma metastases | 57 | Application |
| GSE22154 | Illumina  GPL6947 | 20 melanoma patients with liver and lymphnode metastases | 20 |  |
| GSE54467 | Illumina  GPL6884 | 79 fresh-frozen AJCC stage III melanoma tissues |  |  |
| GSE98394 | Illumina GPL16791 | 51 treatment-naive primary melanoma | 51 |  |
| GSE24551 | GPL5175  GPL1261 | 160 stage II and III CRC tissue samples | 160 |  |
| GSE39582 | Affymetrix GPL570 | 585 CRC tumors | 585 |  |
| GSE17538 | GPL570  GPL11028 | 232 colorectal cancer patients | 232 |  |
| GSE14333 | Affymetrix GPL570 | 290 primary colorectal tumour samples | 290 |  |
| GSE91061 | Illumina GPL9052 | 109 RNASeq samples (58 On-treatment and 51 Pre-treatment) from 65 patients | 109 |  |
| TCGA | | 472 Melanoma patients and 689 colorectal cancer patients | |  |

**Supplementary Table 2A.** **mRNA from purified cell populations : mixtures' proportions.**

| **Mixture ID** | **CD56+ NK cells** | **CD19+  B cells** | **CD66b+ Granulocytes** | **CD3+**  **T cells** | **CD14+ Monocytes** | **HCT116 colon cancer cell line** |
| --- | --- | --- | --- | --- | --- | --- |
| RNA Mixture 1 | 0.0000 | 0.0000 | 0.0000 | 0.0000 | 0.0000 | 1.0000 |
| RNA Mixture 2 | 0.0000 | 0.0000 | 0.0000 | 0.0000 | 0.0000 | 1.0000 |
| RNA Mixture 3 | 0.3521 | 0.0211 | 0.0106 | 0.0880 | 0.1761 | 0.3521 |
| RNA Mixture 4 | 0.1724 | 0.3448 | 0.0069 | 0.0448 | 0.0862 | 0.3448 |
| RNA Mixture 5 | 0.1142 | 0.2283 | 0.1142 | 0.0274 | 0.0594 | 0.4566 |
| RNA Mixture 6 | 0.0506 | 0.0973 | 0.0506 | 0.3891 | 0.0233 | 0.3891 |
| RNA Mixture 7 | 0.0218 | 0.0473 | 0.0218 | 0.1818 | 0.3636 | 0.3636 |
| RNA Mixture 8 | 0.3636 | 0.1818 | 0.0218 | 0.0473 | 0.0218 | 0.3636 |
| RNA Mixture 9 | 0.0233 | 0.3891 | 0.0506 | 0.0973 | 0.0506 | 0.3891 |
| RNA Mixture 10 | 0.0594 | 0.0274 | 0.1142 | 0.2283 | 0.1142 | 0.4566 |
| RNA Mixture 11 | 0.0862 | 0.0448 | 0.0069 | 0.3448 | 0.1724 | 0.3448 |
| RNA Mixture 12 | 0.1761 | 0.0880 | 0.0106 | 0.0211 | 0.3521 | 0.3521 |

**Supplementary Table 2B.** **Proportion of cells measured by single-cell RNA-seq for 10 patients with colon cancer.**

| **donor ID** | **B cells** | **CD4+ T cells** | **CD8+ T cells** | **DCs** | **Monocytes** | **NK cells** |
| --- | --- | --- | --- | --- | --- | --- |
| P0104 | 0.0000 | 0.0000 | 0.0000 | 0.0000 | 0.0000 | 0.1587 |
| P0305 | 0.0187 | 0.0075 | 0.0412 | 0.0300 | 0.5281 | 0.2622 |
| P0309 | 0.0219 | 0.2982 | 0.1946 | 0.0320 | 0.0725 | 0.2140 |
| P0411 | 0.0950 | 0.4276 | 0.2937 | 0.0151 | 0.0032 | 0.0313 |
| P0413 | 0.1592 | 0.2863 | 0.2126 | 0.0110 | 0.0358 | 0.1636 |
| P0720 | 0.3293 | 0.0040 | 0.0040 | 0.0061 | 0.0061 | 0.1071 |
| P0728 | 0.1349 | 0.0064 | 0.0064 | 0.0725 | 0.2774 | 0.0305 |
| P0825 | 0.1175 | 0.3142 | 0.1846 | 0.0028 | 0.0909 | 0.1380 |
| P1212 | 0.0075 | 0.4416 | 0.4096 | 0.0055 | 0.0287 | 0.0137 |
| P1228 | 0.0744 | 0.4094 | 0.3642 | 0.0079 | 0.0165 | 0.0171 |

**Supplementary Table 2C.** **Proportion of cells measured by single-cell RNA-seq for 15 patients with melanoma.**

| **donor ID** | **B cells** | **CD4+ T cells** | **CD8+ T cells** | **Macrophages** | **NK cells** |
| --- | --- | --- | --- | --- | --- |
| Mel04.3 | 0.0256 | 0.2692 | 0.3974 | 0.0256 | 0.0897 |
| Mel102 | 0.0404 | 0.1460 | 0.0994 | 0.0870 | 0.0000 |
| Mel103 | 0.2019 | 0.0473 | 0.1798 | 0.0442 | 0.0000 |
| Mel105 | 0.1282 | 0.1624 | 0.1410 | 0.0299 | 0.0171 |
| Mel106 | 0.1789 | 0.0842 | 0.0421 | 0.0000 | 0.0000 |
| Mel110 | 0.0039 | 0.0426 | 0.0620 | 0.3450 | 0.0000 |
| Mel112 | 0.2832 | 0.1062 | 0.0973 | 0.0354 | 0.0177 |
| Mel116 | 0.2024 | 0.1786 | 0.3452 | 0.0476 | 0.0000 |
| Mel121.1 | 0.0667 | 0.1619 | 0.2476 | 0.1048 | 0.0667 |
| Mel126 | 0.0316 | 0.1368 | 0.5684 | 0.1474 | 0.0105 |
| Mel128 | 0.2472 | 0.1573 | 0.1011 | 0.0225 | 0.0000 |
| Mel129 | 0.0190 | 0.0927 | 0.2763 | 0.2423 | 0.0234 |
| Mel194 | 0.0000 | 0.0924 | 0.4378 | 0.0241 | 0.0000 |
| Mel478 | 0.1753 | 0.3052 | 0.3312 | 0.0000 | 0.0065 |
| Mel98 | 0.0000 | 0.0481 | 0.4630 | 0.0963 | 0.0074 |

**Supplementary Table 2D.** **Proportion of cells measured by single-cell RNA-seq for 19 patients with melanoma.**

| **donor ID** | **B cells** | **CD4+ T cells** | **CD8+ T cells** | **Macrophages** | **NK cells** |
| --- | --- | --- | --- | --- | --- |
| Mel53 | 0 | 0.2985 | 0.1716 | 0.0896 | 0.0746 |
| Mel58 | 0.0168 | 0.1849 | 0.605 | 0.0168 | 0.0336 |
| Mel59 | 0 | 0 | 0 | 0.0143 | 0 |
| Mel60 | 0.4502 | 0.1517 | 0.1517 | 0.019 | 0.0474 |
| Mel65 | 0.0943 | 0.1509 | 0.434 | 0.0189 | 0 |
| Mel67 | 0.2289 | 0.3493 | 0.2892 | 0 | 0.012 |
| Mel71 | 0 | 0.069 | 0.1724 | 0.023 | 0 |
| Mel72 | 0.2612 | 0.3731 | 0.1493 | 0 | 0.0075 |
| Mel74 | 0.1008 | 0.1085 | 0.6667 | 0.0388 | 0.0078 |
| Mel75 | 0 | 0.0454 | 0.9364 | 0.003 | 0 |
| Mel78 | 0.0077 | 0 | 0 | 0 | 0 |
| Mel79 | 0.0902 | 0.0532 | 0.252 | 0 | 0.0012 |
| Mel80 | 0.1181 | 0.2193 | 0.1349 | 0 | 0.0072 |
| Mel81 | 0.016 | 0.0374 | 0.0963 | 0 | 0 |
| Mel82 | 0.0133 | 0.12 | 0.08 | 0.0533 | 0.0267 |
| Mel84 | 0.1724 | 0.1655 | 0.1448 | 0.1448 | 0.0483 |
| Mel88 | 0.0508 | 0.1142 | 0.1175 | 0.1206 | 0.0286 |
| Mel89 | 0.2409 | 0.0818 | 0.2955 | 0.0591 | 0.0023 |
| Mel94 | 0.2025 | 0.1677 | 0.0791 | 0.0063 | 0.0032 |

**Supplementary Table 3.** **All marker genes of eight cell subsets collected from literature research and alive tools.**

| **Cell Type** | **Marker Gene Number** | **Marker Gene** |
| --- | --- | --- |
| B cells | 422 | *AFTPH, AHSP, ANXA3, CNOT1, HLA-DPB1, HLA-DQA1, LSM6, MEFV, MS4A1, NMUR1, PADI4, PYGM, STRN4, TRAF3, TCL6, BLK, WNT16, CD37, CD19, HRH4, CA1, COL19A1, GNG3, PLIN1, SGCA, CD79A, CD79B, ARHGAP17, BTK, CD53, CD72, CIITA, CRX, CXCR5, DCLRE1C, FCER2, HTR3A, IGLL1, KHDRBS2, KIAA0125, LY9, MBD4, MYBL2, P2RY14, POU2AF1, SP140, TCL1A, TCL1B, TLR7, TSHB, VPREB3, CD24, CD40, 1, ACRV1, ACTN2, ADAM20, ADAM21, ADAM30, ADAMTS12, ADAMTS8, ADARB2, ADCY2, ADCY8, AFF2, AFM, AGXT, AHDC1, AICDA, AIPL1, AKAP6, AKAP8L, ALOX15B, ALPL, ANAPC2, ANKMY1, ANKRD34C, AP1M2, AP3B1, APOC3, AQP8, ARG1, ARPP21, ART1, ART4, ASPM, ATF7IP, ATP1B4, AZU1, BAIAP3, BCL2L10, BCL2L11, BMP10, BMP3, BMP8B, BMX, BTBD7, C10orf76, C12orf49, C16orf59, C2orf49, C4BPA, C5orf15, CA14, CACNA1F, CACNA1G, CALY, CAPN3, CASQ2, CCDC81, CCKAR, CCL17, CCR6, CCR8, CCR9, CD180, CD1A, CD1C, CD22, CD3EAP, CDC40, CDK13, CEACAM21, CENPA, CEP55, CEPT1, CER1, CETP, CHAD, CHP2, CHRM2, CHRNA2, CHST5, CLCA4, CLCN1, CLDN14, CLDN17, CLEC1A, CNGB3, CNKSR1, CNR1, CNR2, CNTFR, COLEC10, COQ3, COX6A2, CPA2, CPB1, CR1, CRB1, CRH, CSHL1, CSN1S1, CSNK1G3, CSRP3, CTSG, CUBN, CXCL13, CXorf36, CYLC2, CYP2A7, CYP2C19, DAXX, DAZL, DCC, DDX4, DEF8, DEPDC5, DKKL1, DLX4, DNASE1, DNTT, DPEP3, DPP6, DPYS, DSCR4, DSP, EDF1, EFNA2, EGOT, ESPL1, FBRS, FBXO24, FCAR, FCN2, FCRL2, FGF8, FIP1L1, FLT3, FMO1, FMO6P, FOXM1, FRS2, FRS3, FSCN2, FSCN3, FSHR, FSTL4, GABRA4, GABRA6, GAD2, GCK, GCM1, GDF10, GDI2, GGA2, GH1, GK2, GLYAT, GMFB, GMIP, GNAT2, GNL2, GNRHR, GPR18, GPR25, GPR3, GPR4, GPRC5D, GPX5, GREB1, GRIK3, GRIN2B, GRM6, GTSE1, GYS2, H2AFX, HAMP, HCRTR2, HDAC7, HECW1, HIST1H2BL, HIST1H2BM, HLA-DOA, HNRNPA0, HNRNPL, HP1BP3, HPS4, HSD3B2, HSPA4, HSPB6, HTN3, HTR1B, HTR5A, IFNA1, IFNA2, IFNW1, IKZF3, IL12B, IL17A, IMP4, INHBC, INPP5B, IQCC, ITSN2, BANK1, CD79A, CD79B, FCER2, FCRL2, FCRL5, MS4A1, PAX5, POU2AF1, STAP1, TCL1A, AFF3, BANK1, BLK, BTLA, CCR6, CD180, CD19, CD22, CD37, CD72, CR2, EBF1, FAM129C, FCRL1, FCRL3, FCRLA, HLA-DOB, IGHV5-78, KIAA0125, LINC00926, LOC100507616, LY9, P2RX5, PNOC, POU2F2, S1PR4, SNX22, TLR10, VPREB3, GUSBP11, IGH, IGHG3, IGJ, IGKC, IGKV1D-13, IGLC1, IGLJ3, IGLL3P, IGLV@, IGLV1-44, MZB1, TNFRSF17, TXNDC5, FCRL3, SNX29P1 /// SNX29P2, CLLU1 /// LOC100507616, LOC100129447, ABCB4, CR2, IGHV5-78, IGHM, IGHA1 /// IGHA2 /// IGHD /// IGHG1 /// IGHG3 /// IGHG4 /// IGHM /// IGHV4-31, IGH /// IGHA1 /// IGHA2 /// IGHD /// IGHG1 /// IGHG3 /// IGHG4 /// IGHM /// IGHV3-23 /// IGHV4-31, IGH /// IGHA1 /// IGHA2 /// IGHD /// IGHG1 /// IGHG3 /// IGHG4 /// IGHM /// IGHV4-31, IGHA1 /// IGHA2 /// IGHD /// IGHG1 /// IGHG3 /// IGHM /// IGHV4-31, IGK /// IGKC, IGKV1-17 /// IGKV1-17, IGH /// IGHA1 /// IGHD /// IGHG1 /// IGHG3 /// IGHM /// IGHV3-23 /// IGHV4-31, IGH /// IGHA1 /// IGHA2 /// IGHD /// IGHG1 /// IGHG2 /// IGHG3 /// IGHM /// IGHV4-31, IGLJ3, IGK, IGKC /// IGKV2-28 /// IGKV2-28 /// IGKV2D-28 /// IGKV2D-28, abParts /// IGKC /// IGKV4-1 /// IGKV4-1, IGHA1 /// IGHA2 /// IGHG1 /// IGHG3 /// IGHM /// IGHV3-23 /// IGHV4-31, AL928768.3 /// Ig alpha 1-[alpha]2m /// IGH, IGLC1 /// IGLV3-25 /// IGLV3-25, LOC101929272, IGHD, IGLV4-60 /// IGLV4-60, IGKV1-37 /// IGKV1-37 /// IGKV1D-37 /// IGKV1D-37, IGHA1 /// IGHG1 /// IGHM /// IGHV3-23 /// IGHV4-31, IGKC, IGHG1 /// IGHM, IGHA1 /// IGHD /// IGHG1 /// IGHG3 /// IGHM /// IGHV4-31, IGLC1 /// IGLV3-10 /// IGLV3-10, IGLJ3 /// IGLV3-19 /// IGLV3-19, CKAP2 /// IGLC1 /// IGLJ2 /// IGLJ2 /// IGLJ3 /// IGLJ3 /// IGLJ3 /// IGLV1-44 /// IGLV2-14 /// IGLV@, IGLC1 /// IGLJ3 /// IGLV2-14 /// IGLV@, IGLV1-44, IGLL5, IGH /// IGHA1 /// IGHA2 /// IGHG1 /// IGHG2 /// IGHG3 /// IGHM /// IGHV4-31 /// LOC102725526, IGKV1OR2-108 /// IGKV1OR2-108, AC128677.4 /// CH17-132F21.1 /// IGKV1OR-1 /// IGKV1OR-1 /// IGKV1OR10-1 /// IGKV1OR10-1 /// IGKV1OR2-2, STAP1, PAX5, BMS1P20, CPNE5, RALGPS2, DTX1, KLHL14, IGHG1 /// IGHM /// LOC102725426 /// MIR8071-1 /// MIR8071-2, CKAP2, LINC00926, SNX22, FCRL5, EBF1, IGLC1 /// IGLV9-49 /// IGLV9-49, CKAP2 /// IGLC1 /// IGLJ2 /// IGLJ2 /// IGLJ3 /// IGLJ3 /// IGLJ3 /// IGLV3-1 /// IGLV3-1 /// IGLV@, CKAP2 /// DKFZp667J0810 /// IGLC1 /// IGLJ2 /// IGLJ2 /// IGLJ3 /// IGLJ3 /// IGLJ3 /// IGLV1-36 /// IGLV1-36 /// IGLV1-44 /// IGLV1-50 /// IGLV1-50 /// IGLV@, CKAP2 /// IGLC1 /// IGLV3-1 /// IGLV3-1 /// IGLV@, LOC100130458, LOC100507616, AC079767.4, RP11-138I18.2, CTA-250D10.23, FCRL1, LOC100131043, CXCL12, CCL19, CCL21, CCL25, CCL20, CCL3, AKNA, ARHGAP25, CD2, CD27, CD38, CLEC17A, CLEC9A, CLECL1, FAIM3, FAM65B, GIMAP4, MAP4K1, TNFRSF17, TRAF3IP3, CYBB, ETS1, FAM129C, FCRLA, HDAC9, HLA-DOB, HVCN1, KIAA0226, NCF1, NCF1B, P2RY10, PNOC, SP100, TAGAP, TXNIP, ZCCHC2, CCNA2, CDKN3, CLCN5, ENPP1, FCER1A, STAT5B, TLR9, FCRL4, MYC, RUNX2, SORL1, SOX5, STAT5A* |
| CD4+ T cells | 885 | *SEPT9, CD2, CD3G, CD4, GIMAP6, GLG1, HMOX2, IL7R, ITK, LIMD2, LY9, NAA16, OBSCN, PACS1, PLCL1, RPL14, SNPH, TPP2, TRAF1, ZBTB40, CD40LG, ANKRD55, CA5B, CCR7, LRRN3, MSL3, NPAT, PHF1, RPLP2, RPS6, TMEM30B, TRAF3IP3, TRAT1, PIK3IP1, ANKRD12, MT1E, MAN1C1, PHF20L1, PRMT2, CREBZF, AAK1, ACAP1, ACBD4, APBB1, ARFRP1, ATXN7, BMS1, CABIN1, CASP8, CD226, CD247, CD27, CD28, CD3E, CD5, CD6, CD7, CDK1, CDK10, CEPT1, CHMP7, CLC, CLUAP1, COPS7B, COQ6, CRLF3, CTLA4, CTSW, CUBN, CUL1, DDX31, DDX50, DIDO1, DNAJB1, DPEP2, DSC1, FAM193B, FCF1, FKTN, FNBP4, GIN1, GPSM3, GRAP2, GRK6, GZMM, HAUS3, ICOS, IDUA, IL16, INPP4A, INSL3, IPCEF1, JAK3, KDM3A, KLHL3, KRI1, KRT2, LAIR2, LEPROTL1, MAK, MKL1, MLH3, MLXIP, MTRF1, NCK2, NDFIP1, NOL9, NUDCD3, NUDT9, NUMA1, NUP50, PARP11, PIP4K2B, PLCG1, PLXDC1, POLR3E, POP5, PRMT3, RAB3GAP1, RAPGEF6, RBL2, RBMS1, REV1, RNF216, RNPEPL1, RPA3, RPAP2, RPL38, RPRD2, RXRB, SELPLG, SETD5, SH2B1, SIRPG, SIT1, SLTM, SORCS3, STAP1, SUPV3L1, TATDN2, TBC1D5, TEX264, TNFSF8, TNK1, TSPAN32, TUG1, UBASH3A, USP16, UTP20, VPS52, WDR6, WDR82, ZAP70, ZNF263, ZNF264, ZNF394, ZNF609, ZNF76, ZNF780B, CCR4, HS3ST3B1, IL10RA, FOXP3, TNFRSF4, LAX1, STAT5A, DUSP4, TNFRSF9, IL2RA, STAT5B, BANP, FASLG, STAM, UBE4A, VPS54, ZCCHC8, CCR8, TTN, CCR3, GALNT8, NFATC3, PPM1B, ATG2B, ZFYVE9, SMAD2, SMAD4, KLRC2, IFNG, IL2, MNAT1, SLAMF1, STAT1, EIF2B2, APBB2, CCL4, GGT1, LTA, SYNGR3, TACO1, CXCR6, TBX21, IL12RB1, STAT4, CHD1L, CHD4, CSTF1, LAG3, POLD2, PPM1G, SNRPC, THOP1, CDC123, FIBP, MDC1, TRIM28, GNLY, RUVBL2, NCAPD3, R3HDM1, TMEM39B, UBAP2, CUEDC2, COX10, PSMD3, PKMYT1, PTTG1, KIF20A, RNPS1, TTLL5, NUP205, ZBTB32, HTRA2, WRAP53, WDR18, BST2, CD38, CD70, CSF2, DPP4, IL12RB2, IL22, APOD, ATP9A, BTG3, CMAH, DGKI, DOK5, DUSP5, EGFL6, HBEGF, LRP8, SGCB, ZBTB322, GZMK, IL4, GATA3, GSTA4, SLC25A44, RRAS2, GZMA, CDK2AP1, RGS9, IL5, IL13, MAD2L1, RRM2, BAG2, CEP55, NUP37, NPHP4, GPR15, RAD50, THADA, RNF34, BIRC5, IL26, ADCY1, AHI1, AI582773, ANK1, CDC25C, CDC7, CENPF, DHFR, EVI5, HELLS, LIMA1, MB, MICAL2, NEIL3, PHEX, PTGIS, SLC39A14, SNRPD1, WDHD1, IL1R1, RORC, IL21, IL17RA, IL1R2, STAT3, RORA, IL17A, CA8, KLRB1, MAP4K1, ST8SIA1, TRAC, TRAV9-2, TRIB2, SIK1, FOSB, SH2D1A, BCL6, PVRIG, CD69, ATHL1, BCL11B, CD3D, CHI3L2, CXCL13, CXCR5, DGKA, FAIM3, FZD3, GPR19, ICA1, LAT, LCK, LEF1, MAP9, PASK, PDCD1, PTPRCAP, RGS1, RPL3P7, SLC7A10, TCF7, TRAV13-1, TRAV8-6, TRBC1, TSHR, ZBTB10, CORO7, ATM, GMEB2, SNRPN, ADSL, TFAP4, CYLD, GIMAP4, PURA, DVL1, RPP38, LRIG2, CDKN2AIP, TRADD, TPR, ARID5B, NCK1, SPTAN1, GPR171, INPP4B, MORC2, COL5A3, CBLL1, BMI1, SELL, NUP188, ABCD2, ELP3, USP39, 44084, 44083, ADAT1, ADCYAP1R1, AK1, AKAP3, ALK, ANAPC5, ARHGAP15, ATF7IP, BAG3, BMPR1A, C21orf2, C21orf59, CAMSAP1, CCR6, CD48, CD8A, CD8B, CD96, CDC14A, CGRRF1, CHST5, CNIH4, CRTAM, CRY2, CSNK1D, CXCR3, CYP20A1, DAB1, DCTN6, DDX24, DGCR14, DHX16, DLEC1, DNAI2, DNMT1, DUSP11, DYNLT1, EDC4, EIF2B5, ERN1, ESR2, EXOC1, FASTK, FBXL8, FXYD7, GGA3, GLTSCR2, GOLGB1, GP5, GPR25, GZMH, HAO2, HELZ, HIST1H4F, HMGN4, HNRNPUL1, HUWE1, IKZF1, IL2RB, INPP5E, INTS5, ISCA1, ITGB1BP1, ITIH4, JOSD1, KALRN, KBTBD2, KBTBD4, KIF2A, KLRD1, KLRG1, KRT1, MAN2C1, MCF2L2, MED6, MMP11, MTO1, MYO16, NAP1L4, NCDN, NDRG3, NFRKB, NME6, NR2C1, NUP85, NXF1, OLAH, PCDHA10, PCDHGA9, PIGG, PLCH2, PNMA3, POLR2A, POU6F1, PPWD1, PSD, PSMD2, PTPN4, RAD9A, RAE1, RASA2, RBM5, RPS16, RPS21, RPS6KB1, RRS1, S100B, SARDH, SCNN1D, SEC24A, SFXN1, SGCD, SGSM2, SLC4A5, SLC6A7, SMAP1, SMG5, SNTG2, SOCS3, SPEG, STK11, STUB1, SUPT6H, SYN2, TAB2, TBCC, TCF20, TCF25, THAP11, TNKS2, TNRC6B, TOMM7, TPO, TRAF3IP1, TRIM46, TRMT61A, TSC1, UBQLN2, USP10, USP36, USP4, VPS33A, WDR59, XCL1, XPC, YLPM1, ZBTB1, ZC3H13, ZC3HAV1, ZMYND11, ZNF200, APBA3, CD160, CHST12, COG4, CX3CR1, EWSR1, IKZF3, MAPKAPK5, MRFAP1L1, PSMC5, RNF167, SBF1, SF3B2, TBCD, USP47, ZNF549, CKLF, HBZ, CTBS, CDC42, RABGGTA, GOLGA1, PRKG2, RIC8A, SACM1L, CCR9, OPTN, ZNF354A, PGK1, ABCF1, ABCF3, ABT1, ACADVL, ACTR1B, AHCTF1, AHNAK, AIRE, ALKBH4, AMBRA1, ANAPC2, ANGEL2, ARAF, ARHGEF1, ARHGEF5, ARPC5L, ASB6, ASTE1, ATR, B4GALT3, BCAS2, BIN2, BIN3, BRPF1, BTN2A1, C14orf169, C19orf53, C1orf35, C2orf42, C7orf26, C8G, CACNB1, CALM1, CAPN10, CAPN2, CAPZB, CCDC130, CCDC85C, CCNT1, CCR2, CCR5, CD300A, CD52, CDC37, CDC73, CEP250, CHD8, CIAO1, CLIP1, CNNM2, CNOT1, COLQ, COPB1, COPS6, CROCC, CSNK1G2, CSTF2T, CTBP1, CTDSP1, CTR9, CYTH4, DAXX, DDX18, DDX56, DEFB126, DHX8, DIAPH1, DMWD, DNAJC24, DPP8, DUSP8, DYNC1H1, E4F1, EIF3A, EIF3G, ELAC2, ELK4, EMD, ERAL1, ESYT1, EXOC2, FBXO3, FBXO31, FBXW4, FLT4, FYCO1, GCC1, GIPC1, GIPR, GIT1, GOLGA4, GOLGA7, GORASP2, GPI, GPKOW, GPR52, GPR65, GPR68, GRM3, GSDMD, GTF3C1, GYG1, GZMB, HIST1H3A, HIVEP3, HLA-A, HLCS, HMGXB3, IBTK, IDE, IDH3B, IK, IKBKAP, IL18RAP, IL5RA, IMP3, IRF3, ITGAL, ITGB7, JMJD6, JTB, KIAA0196, KIAA0368, KIAA1109, KIF22, KLHDC4, KLHL11, LIPT1, LTB4R2, TOE1, TUBGCP5, SNX19, KRIT1, CCNT2, DDX5, EZH1, FCN1, PABPC3, RXRG, TPT1, FUBP1, UBA3, USP33, ZBTB11, PRPF38B, ASXL2, THUMPD1, ALG13, FBXO11, ZNF611, NFATC2IP, TMEM123, ZFC3H1, TOMM20, ZNF335, ZNHIT6, DGKA, FOXP3, GCNT4, IL2RA, MDS2, RCAN3, TBC1D4, TRAT1, MIP3, CXCL11, CXCL10, CXCL9, CCL3, CCL5, CCL20, CCL19, CCL21, CCL17, CCL1, CCL22, CCL28, ANLN, BRIP1, BUB1B, CASC5, CCNB1, CCNB2, CCNE2, CKAP2L, DLGAP5, DTL, E2F8, ECT2, ESCO2, EXO1, EXOC6, FBXO5, FIGNL1, HMMR, IARS, KIF11, KIF18A, KNTC1, MASTL, MTHFD2, NCAPG2, NCAPH, NUF2, PRC1, PSAT1, RTKN2, ADAM19, AIM2, COL4A1, SPOCK2, TXK, MALT1, NXN, PLCL2, PNRC1, EPHA4, FYN, IL6R, KLF10, MAL, CASP3, CNOT10, COPB2, DARS, EXOSC9, EZH2, GDE1, IFT74, KLF5, NDUFB9, SHCBP1, XRCC6, B3GAT1, CD200, CD83, CD84, CDK5R1, FGF2, GPR18, ADAM8, CD151, CD53, PTPRC, SPN, TLR6, TNFRSF1A, GPR84, HAVCR2, ICAM1, ICAM3, IGSF6, IL12A, CD68, CD97, EBI3, ITGB4, LTB, METRNL, P2RX5, IRF1, ITGA4, ITGAM, ITGAX, ITGB2, ABP1, C2CD4A, B4GALNT4, C2CD4B, CA2, CCDC65, TRAF3IP2, YBX2, CEACAM3, CHRM3, DOC2B, F12, FURIN, ILDR1, LONRF3, LTK, MOCOS, SH2D6, TNIP2, IL17C, IL17F, IL17RC, IL17RE, IL23A, ASB2, CALD1, CSRP2, DAPK1, DLC1, RBMS3, RNF125, SIGLEC10, SKAP1, TMPRSS3, DNAJC12, DUSP6, GNAI1, HTR2B, LAMP3, NRP2, OSBPL1A, PDE4B, PHLDA1, PLA2G4A, RAB27B, CD34, CD72, GADD45B, WIPF1, TGFB1, TIGIT, TLR10, TLR2, TLR7, TLR8, GEM, IL1RL1, IL9R, MADCAM1, MYH10, NCF2, RCSD1, RYR1, SELE, SELP, SFRP1* |
| CD8+ T cells | 807 | *MAN1C1, CRTAM, BLNK, CCR7, KERA, ZNF208, TREM1, CD8A, CD8B, GPR15, HTR1B, SMCP, NKTR, PSG11, SLC17A4, RRH, SMR3B, CA14, FXYD7, CCDC87, LIN28A, MOGAT2, TNKS2, GJB4, KRT1, CALY, COX4I1, LRRN3, KIAA1109, NGDN, NDUFA1, LEF1, CWF19L1, TPP2, APOBEC3B, CD248, LUZP4, CCR8, HAUS3, RFX2, AMBN, EEF1D, MYL1, NDUFA4, NDUFS5, OMG, SKI, SON, SHFM1, HIST1H3A, HIST1H4F, RNMT, BUD31, GPR52, ZNHIT3, RNF7, JOSD1, BCAS2, RPP38, CGRRF1, JMJD6, RRP8, C19orf53, SETD2, IL21R, MED31, SS18L2, CDK5RAP1, GDAP2, DDX24, USP36, PCIF1, MS4A5, BTNL8, SLC35E1, HIGD2A, CILP, KNG1, PSORS1C2, SCN3A, TNFRSF10C, ITGAM, KLRK1, GNLY, GZMH, PTGDR2, GZMA, PRF1, TBX21, PRDM1, MPO, CD28, CTSW, GZMB, KLRB1, KLRD1, NKG7, KLRF1, RORA, RUNX3, SIGIRR, APOL3, DUSP2, WHDC1, ZBTB16, ADGRG1, AFAP1L2, CCND2, CD38, CHST12, CTLA4, DFNB31, EOMES, FUT8, ITM2A, LAG3, MYO1E, NDFIP2, PARK7, PDCD1, SIRPG, SNX9, CD244, CXCL13, CXCR6, HLA-DRA, IFNG, NAB1, CCL3, VCAM1, HAVCR2, LYST, SARDH, HLA-DMA, LINC00299, ITGAE, MYO7A, RAB27A, CD63, ENTPD1, TNFRSF9, PHLDA1, TIGIT, SNAP47, WARS, CD27, RGS1, TNFRSF1B, CD27-AS1, ACP5, AKAP5, ENTPD1-AS1, TOX, LAYN, RGS2, FKBP1A, MTHFD1, GALM, CSF1, ICOS, MIR155HG, TPI1, CREM, PKM, DUSP4, UBE2F-SCLY, IGFLR1, UBE2F, ID3, CD2BP2, CTSD, STAT3, BST2, RALGDS, TRAFD1, SYNGR2, VAPA, IFI35, MIR4632, YARS, PRKAR1A, IL2RB, HMGN3, MTHFD2, PRDX5, MS4A6A, PTGER4, CORO7, ATM, GMEB2, SNRPN, ADSL, ITK, TFAP4, NAA16, LY9, CYLD, GIMAP4, PURA, DVL1, LRIG2, IL7R, CDKN2AIP, APBB1, IPCEF1, CD247, CD40LG, TRADD, CD3E, TPR, ARID5B, UBASH3A, NCK1, SPTAN1, GPR171, CD5, KDM3A, TRAT1, INPP4B, MORC2, COL5A3, CBLL1, BMI1, SELL, NUP188, ABCD2, ELP3, USP39, SH2D1A, CA5B, 44084, 44083, AAK1, ACAP1, ADAT1, ADCYAP1R1, AK1, AKAP3, ALK, ANAPC5, ANKRD55, ARHGAP15, ATF7IP, ATXN7, BAG3, BMPR1A, C21orf2, C21orf59, CAMSAP1, CASP8, CCR4, CCR6, CD2, CD4, CD48, CD6, CD7, CD96, CDC14A, CHST5, CNIH4, CRY2, CSNK1D, CXCR3, CYP20A1, DAB1, DCTN6, DGCR14, DHX16, DIDO1, DLEC1, DNAI2, DNAJB1, DNMT1, DUSP11, DYNLT1, EDC4, EIF2B5, ERN1, ESR2, EXOC1, FAM193B, FASTK, FBXL8, GGA3, GIMAP6, GLTSCR2, GOLGB1, GP5, GPR25, GZMK, HAO2, HELZ, HMGN4, HNRNPUL1, HUWE1, IDUA, IKZF1, IL2RA, INPP4A, INPP5E, INTS5, ISCA1, ITGB1BP1, ITIH4, KALRN, KBTBD2, KBTBD4, KIF2A, KLRG1, LEPROTL1, LTA, MAN2C1, MCF2L2, MED6, MMP11, MSL3, MTO1, MYO16, NAP1L4, NCDN, NCK2, NDRG3, NFRKB, NME6, NPAT, NR2C1, NUP85, NXF1, OBSCN, OLAH, PARP11, PCDHA10, PCDHGA9, PIGG, PLCG1, PLCH2, PLCL1, PLXDC1, PNMA3, POLR2A, POU6F1, PPWD1, PSD, PSMD2, PTPN4, PTPRCAP, RAD9A, RAE1, RASA2, RBM5, RPL38, RPS16, RPS21, RPS6KB1, RRS1, S100B, SCNN1D, SEC24A, SFXN1, SGCD, SGSM2, SIT1, SLAMF1, SLC4A5, SLC6A7, SMAP1, SMG5, SNPH, SNTG2, SOCS3, SORCS3, SPEG, STK11, STUB1, SUPT6H, SYN2, TAB2, TBCC, TCF20, TCF25, THAP11, TMEM30B, TNFRSF4, TNFSF8, TNRC6B, TOMM7, TPO, TRAF3IP1, TRAF3IP3, TRIM46, TRMT61A, TSC1, UBQLN2, USP10, USP4, VPS33A, WDR18, WDR59, WDR6, XCL1, XPC, YLPM1, ZAP70, ZBTB1, ZC3H13, ZC3HAV1, ZFYVE9, ZMYND11, ZNF200, APBA3, CD160, CDK10, COG4, CX3CR1, EWSR1, HMOX2, IKZF3, MAPKAPK5, MRFAP1L1, PSMC5, RNF167, SBF1, SF3B2, TBCD, USP47, ZNF549, CKLF, HBZ, CTBS, CDC42, FASLG, RABGGTA, GOLGA1, PRKG2, RIC8A, SACM1L, ZNF394, CCR9, OPTN, ZNF354A, PGK1, ABCF1, ABCF3, ABT1, ACADVL, ACTR1B, AHCTF1, AHNAK, AIRE, ALKBH4, AMBRA1, ANAPC2, ANGEL2, ARAF, ARFRP1, ARHGEF1, ARHGEF5, ARPC5L, ASB6, ASTE1, ATR, B4GALT3, BIN2, BIN3, BRPF1, BTN2A1, C14orf169, C1orf35, C2orf42, C7orf26, C8G, CABIN1, CACNB1, CALM1, CAPN10, CAPN2, CAPZB, CCDC130, CCDC85C, CCL4, CCNT1, CCR2, CCR5, CD300A, CD3D, CD3G, CD52, CDC37, CDC73, CEP250, CHD8, CIAO1, CLIP1, CNNM2, CNOT1, COLQ, COPB1, COPS6, CREBZF, CROCC, CSNK1G2, CSTF2T, CTBP1, CTDSP1, CTR9, CYTH4, DAXX, DDX18, DDX56, DEFB126, DHX8, DIAPH1, DMWD, DNAJC24, DPP8, DUSP8, DYNC1H1, E4F1, EIF3A, EIF3G, ELAC2, ELK4, EMD, ERAL1, ESYT1, EXOC2, FBXO3, FBXO31, FBXW4, FLT4, FYCO1, GALNT8, GCC1, GIPC1, GIPR, GIT1, GOLGA4, GOLGA7, GORASP2, GPI, GPKOW, GPR65, GPR68, GPSM3, GRAP2, GRM3, GSDMD, GTF3C1, GYG1, GZMM, HIVEP3, HLA-A, HLCS, HMGXB3, IBTK, IDE, IDH3B, IK, IKBKAP, IL10RA, IL12RB1, IL18RAP, IL5RA, IMP3, IRF3, ITGAL, ITGB7, JTB, KIAA0196, KIAA0368, KIF22, KLHDC4, KLHL11, KRI1, LAIR2, LIPT1, LTB4R2, CERK, TBC1D31, DKK3, NR1D1, TC2N, LST1, ME1, CA2, SLC4A10, CD44, IL23R, DPP4, RORC, JAML, TMIGD2, NCR3, LTK, SESN1, TLE1, IFNGR1, ADAM12, DUSP1, RUNX2, IL18R1, TSPAN15, CEBPD, P2RY14, IL12RB2, NRIP1, SPOCK2, IKZF2, B3GALT2, HPGD, MBOAT1, CTSH, PHACTR2, SYTL2, CCR1, LOC643733, HACL1, IL4I1, TNF, ODF2L, FKBP11, TTC39C, PBXIP1, PRNP, GALC, MKNK1, TMEM71, NFKBIA, OBFC1, EDEM2, PDK3, PNP, CLUAP1, FKTN, RING1, SF1, TSPAN32, PPP1R2, ZNF611, TTN, NELL2, DSC1, NKRF, RNF113A, PRMT2, IL16, MMP19, NFKB1, PCNT, PFN2, MTRF1, CEPT1, RBM34, CBY1, POP5, RAPGEF6, CIAPIN1, CA6, NDUFS2, MYOM1, SDCCAG3, COG2, UTP20, CCDC25, POLR3E, GGNBP2, WDR82, NDFIP1, TMEM41B, PTGDR, SLC1A7, NPRL2, DHX15, HNRNPL, KRT2, PRL, RBL2, RPL37A, SFPQ, SSTR3, ZNF154, PRPF4B, MED17, HNRNPA0, FNBP4, LSM14A, KLHL3, ZBTB11, SHANK1, ZNF639, GJC2, SDAD1, FTO, COPZ1, MKRN2, UBE2Q1, EML3, RWDD3, FAM134C, CCDC53, FLT3LG, AES, ARHGAP8, C12orf47, C19orf6, C4orf15, CAMLG, GADD45A, KLF9, LIME1, MYST3, PF4, PRR5, RBM3, SFRS7, SLC16A7, THUMPD1, TMC6, TSC22D3, VAMP2, ZEB1, ZFP36L2, ZNF22, ZNF609, ZNF91, HAUS3, JAKMIP1, NAA16, TSPYL1, CXCL9, CXCL10, CXCL11, CCL5, MIP3, ADRM1, AHSA1, C1GALT1C1, CCT5, CCT6B, CETN3, SPC25, TIMM13, TIMM8B, TK1, TUBB, TXNDC17, MND1, MPZL1, MRPS16, PCNA, PTRH2, RFC5, CSE1L, EIF2S1, GAL, GEMIN6, GPT2, KIAA0101, ADAM23, ATP2B4, BCL2L11, CD80, CHN1, PYHIN1, RCAN2, SLFN11, ZEB2, F2R, F8, GABARAPL1, GPR183, MSC, APOBEC3H, ARHGAP10, ATP10D, BIRC3, C3AR1, CD55, CFLAR, CMKLR1, DAPP1, DRAM1, EFNA5, FCGR2C, FCGR3A, FCRL6, FGR, GPR114, HAPLN3, HLA-DMB, HLA-DPA1, HLA-DPB1, IFI16, IL15, JAKMIP1, LY96, PLXNC1, PPP4R1, PTPN22, RGS18, SETD7, SH2D1B, SLA2, STXBP1, TLR5, TYROBP* |
| NK cells | 256 | *DNAJB14, IL18RAP, IL2RB, KIR2DL3, KIR3DL2, NCR1, NCR3, PSMD4, SPON2, XCL1, C1QB, CALY, CD37, CLEC10A, CRTAM, CSF2RB, CX3CR1, GZMB, IL7R, KNG1, MAN1C1, PRR5L, SYT17, CSF3, FGFBP2, GNLY, KLRF1, AKR1C3, PTGDS, PRSS23, CD160, KIR3DS1, CD244, CHRNE, FASLG, PTGDR, RGS9, TBX21, TKTL1, 1, 44081, AGK, ALG13, AMZ2, ANKRD11, ARPC5L, ASTE1, BAD, BRD2, C1orf174, CCL4, CD247, CDKN2AIP, COQ10B, CTSW, DNAJC2, DR1, FBXW4, FIP1L1, GGPS1, GIPR, GNA13, GOLGA4, GPATCH8, GRIK4, GTF3C1, GZMH, GZMM, HELZ, HIPK1, HIST1H3A, HNRNPL, IFNG, IL21R, KLRD1, KLRG1, KPNB1, LAG3, LEMD3, LIM2, LTA, MAP3K7, MAPK1, MED1, MGAT2, MLH1, NEK1, NFE2L2, NKG7, NMUR1, OSBPL7, PJA2, PPP2CA, PRDM2, PRDX6, PRF1, PRKAG1, PTPN4, RAB14, RBM25, RBM39, RSRC2, SACM1L, SBF1, SF3B4, SON, STAG2, STX8, SUPV3L1, TBCC, THAP1, TNFSF11, TSPYL1, TSTD2, UBE2Q1, WBP11, WDR45, YAF2, ZBTB1, ZBTB39, ZCCHC11, ZMYND11, ZNF264, ZNF426, DUSP4, FOXJ1, MPPED1, PLA2G6, RRAD, BG255923, MADD, 43896, MUC3B, NIBP, KIR3DL1, KIR2DS1, KIR3DL3, EDG8, FLJ20699, KIR2DS2, KIR2DS5, TMEPAI, XCL2, BCL2, FUT5, SPN, ZNF205, ADARB1, AF107846, AL080130, ALDH1B1, ARL6IP2, CDC5L, FGF18, FZR1, GAGE2, LDB3, LOC643313, LOC730096, MAPRE3, MCM3AP, MRC2, NM_014114, NM_014274, NM_017616, PDLIM4, PRX, RP5-886K2.1, SLC30A5, SMEK1, TBXA2R, TCTN2, TINAGL1, ZNF528, ZNF747, CLIC3, FGFBP2, GNLY, GNPTAB, KLRF1, NCR1, NMUR1, S1PR5, SH2D1B, KIR2DL1, KIR2DL2, KIR2DL3, KIR2DL4, KIR2DL5A, KIR2DS1, KIR2DS2, KIR2DS3, KIR2DS5, KIR3DL1, KIR3DL2, KIR3DL3, KLRC2, KLRC3, KLRC4, KLRD1, PRF1, SAMD3, TBX21, SH2D1B, KIR3DL1 /// KIR3DL2, KIR2DL4, CCL20, CCL8, CCL7, CCL13, CXCL12, CCL5, IL8, CXCR3, CXCL9, CXCL10, CX3CL1, AKT3, AXL, CDH2, CYTH1, PIK3CG, PILRA, PLCG2, SIGLEC7, SIGLEC9, GRB2, LILRB5, LST1, MAPK4, NOTCH3, C11orf75, DYNLL1, HCP5, HDGFRP2, KRT86, MLST8, MYL6B, TAX1BP3, AKR7A3, CLTB, FAM27A, GLS2, GPRC5C, GRIN1, PORCN, PQBP1, PSMC4, TEX264, UPP1, HLA-E, KIR2DS4, KLHL21, KRT80, MPL, PHRF1* |
| DCs | 245 | *BLVRB, C1QA, C1QB, CSF1R, CXCR3, FGL2, GZMB, IL21R, KCNA5, KCNC3, KCTD5, LILRB4, LMAN2L, NFKB1, PLD2, PTCRA, PTGIR, SIGLEC1, SLAMF8, SLC15A3, SYT17, VAV2, CA8, CALY, CRTAM, CYP4F3, GATA3, ITIH4, PRR5L, ST8SIA1, TNFRSF10C, CCL13, CCL17, CCL18, CCL22, CD1B, CD1E, CD86, CLIC2, FPR3, MMP12, TREM2, PLA2G7, TMEM255A, 1, ACHE, ALCAM, ALDH1A2, ALOX15, ALOX15B, APOC3, ARL8B, BCL2L11, BCL2L13, CACNB1, CAMK1G, CCDC81, CCL19, CCL23, CCL24, CCL8, CCR2, CCR7, CD1A, CD1C, CD209, CD2AP, CD80, CD9, CELA2A, CEP350, CLEC10A, CSHL1, CUL1, CUX2, CXCL13, DNASE1L3, DPYS, ETV3, F13A1, FBXL4, FCER2, FKBP2, FLT3, FUT7, GRIN1, GRSF1, GUCA1A, HCRTR2, HIST1H2BB, HK3, HLA-DQA1, HPD, HPS5, HS3ST2, IDH3A, IL12B, IL3RA, IRF4, KCNK10, KCNK13, KCNN1, LOR, LRRC36, MAP3K13, MAP3K6, MAPKAPK2, MCF2, MPHOSPH6, MS4A4A, MS4A6A, MYBPC1, NAGPA, NECAP2, NXPH3, P2RY14, PRRG2, PTGES2, RAB8A, RNF2, RPL3L, RRP1B, RUNX2, SAMSN1, SCT, SLAMF1, SLC12A3, SLC30A4, SLCO5A1, SLITRK3, SNX11, SPCS1, SPIB, SPINT2, STAB1, SUZ12, TACR1, TACSTD2, TBC1D13, TDRD7, TFEC, TLR7, TMEM131, TMSB10, TNFRSF4, TRAF1, TSPAN13, TXN, UBE2Z, ZNF221, CCL1, EBI3, LAMP3, OAS3, INDO, HSD11B1, NPR1, PPFIBP2, CARD9, CH25H, PPARG, ABCG2, CTNS, FABP4, FZD2, GSTT1, NM_021941, NUDT9, PREP, RAP1GAP, SLC26A6, SLC7A8, TM7SF4, VASH1, C1QC, CCDC88A, CCL3L1, CCL3L3, CD300E, HLA-DQA2, INHBA, LGMN, NGFR, PDGFRL, PDPN, PRKAR2B, SIGLEC5, SLAMF9, THBD, TNFAIP2, TTYH2, UBD, VCAM1, CLEC1A, CLEC4C, CLEC5A, CSF2RA, FAM49A, FCGR1A, FN1, FSTL1, GPR109B, GPX3, ATP6V1A, CHST11, CSF1, DPYD, FNDC3B, TGM2, TIMD4, GRP, IL1RN, INPP5F, LPL, LPXN, MMD, PLAU, PLCB2, RAB38, RDX, RRAGD, ATP5O, CBX1, DNAJC15, ENSA, FOXN3, PRCP, SSB, TPMT, CBX6, DAB2, DDX17, HIGD1A, SIRPA, SPCS3, TMED2, TMX1, UGCG, ZDHHC17, MAGED1, NUCB2, OFD1, OGT, PDIA4, SERTAD2* |
| Macrophages | 364 | *ARPC4, ATP6V0E1, BPI, C1QA, C1QB, CAMP, CHIT1, CLEC5A, CLIP1, CSF1R, CYBB, FGR, GGA1, GRB2, IFNAR1, IGSF6, IL17RA, LILRA2, MARCO, MMP8, MS4A6A, OTUD4, PSME1, RENBP, CD14, FCGR1A, TFRC, CCR5, CD163, CD86, CD80, CCL2, APOC1, CD300C, F13A1, FPR3, HAMP, IL1B, LILRB4, MSR1, SIGLEC1, VSIG4, SDS, FCER1G, HK3, SIGLEC7, TYROBP, MS4A4A, C3AR1, 1, 44084, ABCD1, ABI1, ABTB2, ACADVL, ACP2, ACSM5, ACTR10, ACTR2, ACTR3, ADAMDEC1, ADCK2, ADCY3, ADO, ADRA2B, AFG3L2, AGGF1, AGPS, AKR7A2, ALCAM, ALDH9A1, ALG9, ALK, ANGPT4, ANKFY1, ANXA11, ANXA2, AP1B1, AP1M2, AQP8, ARFGEF2, ARHGEF11, ARL8B, ARSB, ATOX1, ATP2A2, ATP2C1, ATP6AP2, ATP6V0A1, ATP6V0C, ATP6V0D1, ATP6V1A, ATP6V1C1, ATP6V1D, ATP6V1E1, ATP6V1F, ATP6V1H, BAG3, BAIAP2, BCAP31, BCKDK, BLVRA, BTBD1, C10orf76, C12orf4, C12orf49, C16orf62, C7orf25, CALR, CANX, CARD14, CCDC47, CCDC85C, CCDC88A, CCL1, CCL18, CCL19, CCL22, CCL24, CCL7, CCL8, CCR1, CD164, CD48, CD52, CD63, CD81, CD84, CD9, CDS2, CECR5, CEPT1, CETN2, CHD9, CIAO1, CIR1, CLCN7, CLEC4E, CLPB, CLTC, CMKLR1, CNIH4, COL4A3BP, COMMD8, COMMD9, COQ2, CORO7, COX15, COX5A, COX5B, COX7B, COX8A, CPNE6, CRYBB1, CSF1, CXCL9, CYBA, CYC1, CYFIP1, CYP19A1, DAGLA, DBI, DERA, DHX57, DLAT, DNAJC13, DNASE1L3, DNASE2B, DOT1L, ECHS1, EFR3A, ELK1, ELOVL1, EMILIN1, ERP29, EXOC1, EXOC5, FAM32A, FANCE, FDX1, FEZ2, FH, FKBP15, FLT1, FOLR2, FPR2, FTL, G6PC3, GABARAP, GLB1, GLRX2, GORASP1, GP1BA, GPD1, GSTO1, GUCA1A, GUF1, HADHB, HAUS2, HCCS, HEXA, HEXB, HIGD2A, HMGCL, HPS1, HS3ST2, HSD17B12, HSPB7, HSPH1, HTT, HYAL2, IARS2, IBTK, IL10, IL12B, IPPK, ITGAE, ITGAX, ITGB1BP1, KCMF1, KCNJ1, KCNJ5, KCNK13, KCNMB1, KCTD5, KIAA0196, KIFC3, KLHL12, LAIR1, LAMP1, LDHAL6B, LILRB1, LILRB5, LIMD2, LONP1, LONRF3, LY86, M6PR, MAPK13, MAPKAP1, MDH1, MFN1, MFSD7, MGST3, MKL2, MLX, MMP19, MRM1, MRPL12, MRPL40, MRS2, MT2A, MTHFR, MTMR14, MUL1, C1QC, CD14, CD163, CD300C, CD300E, CSF1R, F13A1, FPR3, HAMP, IL1B, LILRB4, MS4A6A, MSR1, SIGLEC1, VSIG4, ADORA3, AOAH, ARRB2, ATP8B4, BCL2A1, C1orf54, C1QA, C1QB, C2, C3AR1, C5AR1, CCR1, CCRL2, CD300A, CD4, CD68, CD74, CD86, CECR1, CLEC7A, CMKLR1, CTSB, CTSS, CYBB, CYTH4, DPYD, EMR2, FCER1G, FCGR1A, FCGR1B, FCGR2A, FCGR3B, GPNMB, HK3, HLA-DRB6, IFI30, IGSF6, ITGAM, ITGAX, ITGB2, LAIR1, LAPTM5, LIPA, LY96, MAN2B1, MFSD1, MNDA, MS4A4A, MS4A7, MYO1F, NCKAP1L, NPL, NR1H3, PLA2G7, PLEKHO2, SCPEP1, SLAMF8, SLC15A3, SLC31A2, SLCO2B1, SNX10, SPI1, TBXAS1, TLR8, TMEM140, TNFAIP2, TNFRSF1B, TNFSF13B, TRPV2, TYMP, TYROBP, CXCL14 , CCL20 , CCL4, AIF1, CCL14, CCL26, CD300LB, CNR1, CNR2, CPM, CSF3R, ENG, FCAR, IGF1, IL34, L1CAM, LILRA1, LILRA5, LRP1, MS4A7, MS4A8B, TREM1,TREML1* |
| Monocytes | 331 | *IL7R, APOC3, ADCY8, CLCA4, CPA2, DPP6, HSPB6, KNG1, KLRK1, GZMH, KLRB1, SGCA, CA1, TLR8, CYBB, IGSF6, KLRF1, CYP4F3, CLEC10A, FOLR2, LTBR, MAP3K2, NUP214, OSBPL11, RTN3, SERP1, TBK1, SOCS3, ASGR2, SH3BP2, RHOT1, HK3, IRAK3, CD33, MS4A6A, VCAN, 1, AATK, ABCB7, ACAP2, ADIPOR1, AGFG1, AHNAK, AIF1, AKAP13, AKAP8, ANKS1A, ANXA1, AP1G1, APAF1, APBB3, ARF5, ARL8B, ARNT, ATG3, ATP6V0D1, AZIN1, BCL10, BCL2L11, BEST1, BNIP2, BPI, BTAF1, BTK, C3AR1, CALCOCO2, CAMKK2, CAPN2, CAPN3, CAPNS1, CARS2, CASP5, CAST, CBX6, CCR2, CD101, CD163, CD1E, CD244, CD300C, CD4, CD48, CD93, CDC40, CDK9, CEACAM4, CECR5, CEPT1, CFP, CLEC1A, CLEC3B, CLEC4A, CLEC4E, CLEC5A, CLIP1, COL4A3BP, COMMD9, COQ2, CSF1R, CSF3R, CSNK1A1, CTBP2, CUL5, CX3CR1, CXCR2, CXorf21, CYSLTR2, DCTN4, DDX21, DDX3X, DENND1A, DHX57, DHX8, DLG4, DNAH17, DNAJC13, DOK2, DOK3, DPEP2, EIF1B, EIF4E2, ELL, ETF1, ETV3, EWSR1, F13A1, FAM32A, FBXL5, FBXO11, FCAR, FCER1A, FCER1G, FCN1, FGL2, FGR, FKBP15, FNDC3A, FOLR3, FPR2, GABARAP, GALNT3, GGA1, GIT2, GNA13, GNMT, GPR162, GPR183, GRPEL1, H2AFY, HADHA, HCK, HIC1, HIF1A, HIPK1, HNRNPU, HRH2, HSPA6, HUS1, IL10RA, IL17RA, IMPDH1, IQGAP1, IQSEC2, JMJD1C, JMJD6, KCNC3, KCNMB1, KDM6B, KIAA1033, KLHL18, KSR1, LILRA1, LILRA2, LILRA5, LILRB1, LILRB2, LILRB3, LRRFIP1, LST1, LY86, LYL1, MAN2C1, MAP2K1, MAP3K11, MAP3K3, MAPK14, MAPK6, MARCO, MARK3, MBOAT7, MED13L, MEFV, METTL9, MIOS, MMP17, MNDA, MNT, MPHOSPH6, MS4A4A, MTF1, MTHFR, MTMR14, MTMR3, MYO1F, NCF4, NCOA4, NDST2, NEK4, NKIRAS2, NPLOC4, NSFL1C, NUBP1, OGFR, OSM, P2RY13, PADI4, PANK2, PCTP, PDE6H, PGGT1B, PGLS, PHKG2, PHLDA2, PIAS1, PIKFYVE, PILRA, PLAA, PLD2, PLEK, PLP2, PNP, POU2F2, PPM1A, PPM1F, PPP1CB, PRKACA, PSTPIP1, PTCH2, PTEN, PTGIR, PTP4A2, PTPN18, QKI, RAB5A, RABGEF1, RARA, RBM41, REEP4, RELA, RETN, RGS19, RHOA, RHOG, RIN2, RIOK3, CD300C, CD300E, CECR1, CLEC6A, CPVL, EGR2, EREG, MS4A6A, NAGA, SLC37A2, AGTRAP, AIF1, C10orf54, CD14, CD300LF, CD33, CD93, CTSD, EMILIN2, FCN1, FES, FGR, GNS, GRN, HCK, HMOX1, KIAA0930, LILRA6, LILRB2, LILRB3, LRRC25, LST1, NFAM1, NOTCH2, PILRA, PLXDC2, PRAM1, PSAP, PYCARD, RHOG, SERPINA1, SLC7A7, TGFBI, THEMIS2, TIMP2, TPP1, VCAN, MIF, NA, IL8, CCL2, CCL8, CCL7, CCL13, CCL12, CX3CL1, ACTR3, ANXA5, ARPC2, ATP6V1B2, BASP1, CD300LF, UBE2D3, LITAF, OLR1, RAB1A, DAZAP2, EIF4A1, EIF4G2, EMP3, FTL, SAT1, SDCBP, SRGN, TEK, TMBIM6* |
| Neutrophil | 225 | *IL18RAP, BMX, BTNL8, CASP5, CLC, CXCR1, CXCR2, FCGR3B, FPR2, HSPA6, MEFV, PADI4, S100A12, TREML2, TRPM6, SIGLEC5, CREB5, ALPL, AATK, CEACAM3, CSF2RB, CSF3R, FBXO38, MAK, PAK2, TOP1, UBXN2B, VNN3, BEST1, CA4, CAMKK2, CEACAM8, CLEC4E, CYP4F3, DHX34, ELL, FCAR, FFAR2, GCC1, HBB, HRH4, LILRA1, LILRA2, LILRB2, MED13L, MMP25, MTMR3, NFYA, NMI, P2RY13, PGLYRP1, PTEN, RMND5A, SDF2, SLC12A1, SLC19A1, SPAG9, TREM1, TRIM25, UBE2B, WDFY3, WWP2, 1, ACAP2, APAF1, BTN2A1, CBL, CIR1, DDX3X, GPR27, HERC3, IP6K1, KSR1, LMTK2, NDEL1, NRBF2, SLC25A44, TECPR2, TGM3, TMEM185B, TMUB2, TOX4, TTLL4, UBE2D1, UBN1, USP15, ZDHHC18, FPR1, BST1, MME, TNFRSF10C, CD93, CRISPLD2, DYSF, FLJ11151, FPRL1, G0S2, HIST1H2BC, HPSE, IL8RA, IL8RB, KCNJ15, KIAA0329, MGAM, PDE4B, SLC22A4, SLC25A37, TNFRSF, CNTNAP3, CXCR1, CYP4F3, FFAR2, HIST1H2BC, HIST1H3D, KY, MMP25, PGLYRP1, SLC12A1, TAS2R40, ACSL1, ALPK1, AQP9, BASP1, BCL6, CD97, CEP19, CFLAR, CSF3R, CXCR2, DENND5A, DYSF, FAM65B, FCGR2C, FPR1, GLT1D1, GPR97, IFITM2, IL17RA, KCNJ2, KIAA0247, LILRA2, LIMK2, LINC01002, MGAM, MOB3A, NAMPT, NCF4, PADI2, PHC2, PHF21A, PLXNC1, PREX1, RALB, RNF149, S100A8, S100A9, SLC25A37, SNORD89, SSH2, STAT3, STAT5B, THBD, TLR2, TLR4, TMEM154, TNFRSF1A, LOC101928717 /// SLC19A1, LINC00528, CTB-167B5.2, NFE4, LOC100289061, IRAK3, NHSL2, MIR7109 /// PISD, ORM1, PLXNC1, TLE3, CYP4F2 /// CYP4F3, LOC254896 /// TNFRSF10C, MEGF9, ARHGAP26, HAL, DOCK5, STEAP4, SLC12A6, LRRC4, DGAT2, NATD1, LRG1, ABTB1, CMTM2, PROK2, KIF13A, PHOSPHO1, RP11-44F14.8, SLC26A8, PCBP1-AS1, NPL, CLEC7A, SKAP2, PLIN5, FAM212B /// LOC101928718, REPS2, PI3, CXCL2, CCL3, IL8, CCL4, CXCL9, CXCL10, CXCL11, CCL17, CXCL1, CXCL5, AMPD2, CAMP, EMR4P, TNFSF14, TREML4, VNN2, XPO6, GPR77, MAEA, SEC14L1, SEPX1* |

**Supplementary Table 4. The shared and different signature genes between colorectal cancer and melanoma.**

| **Cell Type** | **Types** | **Signature Genes** |
| --- | --- | --- |
| B cells | Shared genes  (7) | *BLK, CD19, CD79A, CD79B, IGLL1, TCL1A, TLR7* |
|  | CRC_genes  (17) | *TNFRSF17, EBF1, HLA-DOB, PNOC, ARHGAP25, P2RY10, TLR9, BANK1, KLHL14, CPNE5, FCRL2, FCRL5, FCRL4, FCRLA, CLECL1, LINC00926, NCF1* |
|  | SKCM_genes  (8) | *MS4A1, GNG3, SGCA, CD53, CD72, HTR3A, VPREB3, AICDA* |
| CD4+ T cells | Shared genes  (27) | *ANK1, CD69, CHI3L2, DGKA, GATA3, IL12RB2, JAK3, LCK, NPAT, RGS1, SELPLG, STAT4, STAT5B, HS3ST3B1, CD226, PASK, ANKRD12, STAP1, ZBTB32, LAT, PNMA3, FOXP3, LAX1, BCL11B, PVRIG, LIMD2, PIK3IP1* |
|  | CRC_genes  (38) | *CD40LG, CD72, CCR4, CCR8, FYN, GPR18, GPR19, IL2RA, IL6R, IL9R, TNFRSF9, ITGA4, ITGB2, LTB, MAL, CD200, P2RX5, PDCD1, PLCL1, PTPRC, STAT5A, TXK, WIPF1, SOCS3, AIM2, TLR6, PLCL2, ASB2, LRRN3, RNF125, PARP11, PLXDC1, MAN1C1, HIVEP3, ANKRD55, TRIM46, SIGLEC10, RCSD1* |
|  | SKCM_genes  (58) | *TRAF1, AAK1, ACBD4, CUBN, GPSM3, GRAP2, IL16, INSL3, KLHL3, KRT2, LAIR2, MLH3, MLXIP, NOL9, SORCS3, TNK1, TSPAN32, ZNF780B, TTN, CCR3, NFATC3, IL2, GGT1, SYNGR3, IL12RB1, CSF2, DPP4, IL22, EGFL6, IL4, IL5, IL13, IL26, MB, MICAL2, PHEX, PTGIS, IL1R1, RORC, IL21, IL1R2, IL17A, MAP4K1, SIK1, FOSB, MAP9, SLC7A10, TCF7, TSHR, ZBTB10, TFAP4, COL5A3, ADCYAP1R1, DAB1, ERN1, FXYD7, ARHGEF5, DEFB126* |
| CD8+ T cells | Shared genes  (9) | *RUNX3, PTGER4, ZBTB16, EOMES, ITM2A, AKAP5, TOX, APOL3, KIAA1109* |
|  | CRC_genes  (33) | *ABCD2, CD8A, CD8B, LYST, TSC22D3, GPR183, FLT3LG, HLA-DPB1, IFI16, INPP4A, POU6F1, RAB27A, ATXN7, IL18R1, SLC16A7, SPOCK2, ZEB2, PLXNC1, CA5B, IKZF2, PTPN22, PBXIP1, IGFLR1, SLA2, SLFN11, JAML, TC2N, TTC39C, TMIGD2, TMEM71, HAPLN3, PYHIN1, JAKMIP1* |
|  | SKCM_genes  (34) | *BLNK, HTR1B, SMCP, RRH, CCDC87, MOGAT2, GJB4, CALY, CD248, RFX2, AMBN, MYL1, GPR52, CILP, TNFRSF10C, ITGAM, PTGDR2, PRDM1, MPO, DUSP2, CCND2, SNX9, CXCL13, HAVCR2, LINC00299, MYO7A, TIGIT, GIPR, TNFRSF1B, RGS2, GALM, SYNGR2, CCR6, ATR,* |
| Dendritic cells | Shared genes  (9) | *CD1B, CD1C, CD1E, CLIC2, DNASE1L3, IL3RA, CCL17, CD209, SLAMF8* |
|  | CRC_genes  (6) | *C1QC, FABP4, THBD, SAMSN1, FAM49A, SLAMF9* |
|  | SKCM_genes  (19) | *SLAMF8, CCL17, CD1B, CD1E, CLIC2, CD1C, CD209, DNASE1L3, IL3RA, SAMSN1, FABP4, C1QC, SLAMF9, THBD, FAM49A* |
| Macrophages | Shared genes  (9) | *CD14, CHIT1, CCR1, CMKLR1, SDS, VSIG4, SIGLEC7, ADAMDEC1, HAMP* |
|  | CRC_genes  (9) | *APOC1, CPM, CSF1, ENG, MMP19, CCL8, CCL18, HS3ST2, MS4A7* |
|  | SKCM_genes  (10) | *CAMP, FCGR1A, MSR1, TYROBP, CCL7, CD84, CPNE6, CXCL9, CYBA, CYP19A1* |
| Monocytes | Shared genes  (13) | *ASGR2, CD33, FCN1, HCK, CFP, DOK2, LILRA1, IRAK3, CLEC4A, TLR8, DOK3, CXorf21, LILRA5* |
|  | CRC_genes  (13) | *MNDA, MYO1F, NCF4, PLEK, POU2F2, SRGN, PSTPIP1, QKI, LILRB2, CLEC4E, PILRA, RETN, CD300LF* |
|  | SKCM_genes  (18) | *CA1, FOLR2, VCAN, AIF1, CD101, CD93, LYL1, CEACAM4, FCER1A, FGL2, FOLR3, GPR183, KCNMB1, KDM6B, KSR1, LILRB1, LST1, LY86* |
| NK cells | Shared genes  (5) | *KIR2DL3, KIR3DS1, LIM2, PTGDR, NCR1* |
|  | CRC_genes  (11) | *KIR2DL1, KIR2DL4, KIR2DS1, KIR2DS4, KIR2DS5, KIR3DL1, PIK3CG, FGFBP2, KIR3DL3, SH2D1B, KIR2DS2* |
|  | SKCM_genes  (6) | *NCR3, PRR5L, HIPK1, NMUR1, PRDM2, TNFSF11* |
| Neutrophils | Shared genes  (5) | *ALPL, FCGR3B, FFAR2, VNN3, MMP25* |
|  | CRC_genes  (8) | *CAMP, FPR1, VNN2, TREM1, PROK2, CLEC7A, CMTM2, LINC00528* |
|  | SKCM_genes  (14) | *CXCR1, S100A12, TREML2, TRPM6, SIGLEC5, CREB5, CEACAM3, CA4, CEACAM8, CYP4F3, HBB, P2RY13, PGLYRP1, TGM3* |
